# Supplementary material for: The Women’s Wellness with Type 2 Diabetes Programme: Feasibility of an online peer support and goal-setting intervention for midlife women
Source: PLoS One. 2026 Mar 23;21(3):e0345517. doi: 10.1371/journal.pone.0345517 (PMC13008096; doi:10.1371/journal.pone.0345517)
Supplement: S1 Table — (DOCX) [file pone.0345517.s001.docx]

**Supplementary File 1: Participants read the Components from the Women’s Wellness with Type 2 Diabetes Programme eBook.**

| **Step 1**  **PREPARATION & CHANGING LIFESTYLE** | **Week 1 – Preparation** | Day 1 | A refresher about type 2 diabetes |
| --- | --- | --- | --- |
|  |  | Day 2 | Your feelings about diabetes |
|  |  | Day 3 | Coming to terms with your diabetes  Treatment and what it means for you  Illness – what to do when you feel unwell |
|  |  | Day 4 | Starting to think about routines and balance in your life  Your self-care plan |
|  |  | Day 5 | Overcoming obstacles to change |
|  |  | Day 6 | Your goals for health |
|  |  | Day 7 | Reflection |
|  | **Week 2- Changing Lifestyle** | Day 1 | Healthy eating with type 2 diabetes  Water |
|  |  | Day 2 | Understanding more about glucose |
|  |  | Day 3 | Alcohol and smoking |
|  |  | Day 4 | Healthy shopping and meal planning |
|  |  | Day 5 | Regular physical activity and exercise |
|  |  | Day 6 | Stretching and flexibility  Balance  Strengthening the pelvic floor |
|  |  | Day 7 | Preparing for the week ahead  Week 3 exercise schedule |
|  | **Week 3 –Changing Lifestyle** | Day 1 | Changing your eating behaviours |
|  |  | Day 2 | Strength training exercise  Program 1: Exercises without equipment |
|  |  | Day 3 | Stress and you |
|  |  | Day 4 | Diabetes distress  Strategies for managing stress |
|  |  | Day 5 | Better sleep  Memory and thinking |
|  |  | Day 6 | Goal setting |
|  |  | Day 7 | Review  Review of week 3  Week 4 exercise schedule |
| **STEP 2: ESTABLISHING HEALTHY LIFESTYLE HABITS** | **Week 4-Healthy weight** |  | Healthy weight week  Review of week 4  Week 5 exercise schedule |
|  | **Week 5- Strong Bones** |  | Strong bones week  Osteoporosis prevention  Getting enough calcium and vitamin D  Program 2: Exercises with dumbbells or resistance bands  Program 3: Exercises in a fitness centre  Review of week 5  Week 6 exercise schedule |
|  | **Week 6- menopause** |  | Let’s talk about menopause  Expectations  Menopausal symptoms checklist  Managing menopausal symptoms  Sexuality and menopause  Let’s review the steps you have taken  Review of week 6  Week 7 exercise schedule |
| **STEP 3: MAINTAINING HEALTH FOR ILLNESS PREVENTION** | **Week 7 –Healthy Heart** |  | Healthy heart week  Review of week 7  Week 8 exercise schedule |
|  | **Week 8-Diabetes Complications Prevention** |  | Diabetes complications prevention week  Your regular tests and check-ups  Preventing complications of diabetes  Short- and long-term complications  Review of week 8  Week 9 exercise schedule |
|  | **Week 9 –Cancer Prevention** |  | Cancer prevention week  Goal setting  Review of week 9  Week 10 exercise schedule |
| **STEP 4: BECOMING INDEPENDENT: REVIEW, MOTIVATION AND LOOKING FORWARD** | **Week 10 -Review** |  | Reviewing what you have learned  Review of week 10  Week 11 exercise schedule |
|  | **Week 11- Motivation and change** |  | Motivation and change  Overcoming obstacles to change  Review of week 11  Week 12 exercise schedule |
|  | **Week 12 –Goal Setting** |  | Looking forward – goal setting  Review of week 12  Final word |
