# Supplementary material for: The Women’s Wellness with Type 2 Diabetes Programme: Feasibility of an online peer support and goal-setting intervention for midlife women
Source: PLoS One. 2026 Mar 23;21(3):e0345517. doi: 10.1371/journal.pone.0345517 (PMC13008096; doi:10.1371/journal.pone.0345517)
Supplement: S1 File — (PDF) [file pone.0345517.s003.pdf]

PROTOCOL

1. Full title: Women's Wellness Type 2 Diabetes Programme: A single arm trial of a Newly Designed Intervention for Midlife Women
2. Trial Registry: **King's College London Ethics**: Full Approved Reference Number: HR/DP-23/24-34435
3. Research Team

a. Principal Investigator

I. Deniz Bozkurt, PhD student, Florence Nightingale Faculty of Nursing, Midwifery and Palliative Care, King's College London. deniz.bozkurt@kcl.ac.uk

b. Supervisors:

I. Jackie Sturt, Professor of Behavioural Medicine in Nursing, Florence Nightingale Faculty of Nursing, Midwifery and Palliative Care, King's College London. Jackie.sturt@kcl.ac.uk

II. Maria Duaso (PhD), Senior Lecturer in Care in Long Term Conditions, Florence Nightingale Faculty of Nursing, Midwifery and Palliative Care, King's College London. maria.duaso@kcl.ac.uk

III. Iliatha Papachristou Nadal (PhD), Lecturer in International Healthcare (R&T), Florence Nightingale Faculty of Nursing, Midwifery and Palliative Care, King's College London. iliatha.papachristounadal@kcl.ac.uk

4. Research site: United Kingdom
5. Study Sponsor: King's College London, United Kingdom
6. Study Funder: Ministry of Education in Turkey
7. Trial Management

The trial will be managed by the principal investigator (DB) and her academic supervisors, meeting monthly via face to face or Microsoft Teams during the period of intervention delivery and data collection. Every six months DB will be responsible for the day-to-day administration of the study and reporting to the academic supervisors through emails and monthly supervision meetings. All documents will be prepared and kept at the study site. The principal investigator will be responsible for the recruitment, data entry and analysis.

8. Background rationale of the study

a. Type 2 Diabetes

The incidence of type 2 diabetes (T2DM) has increased significantly in recent years, which has a negative impact on people's lives on a daily basis. According to International Diabetes Federation (IDF) (2021), the prevalence of the number of people living with diabetes is estimated at 643 million by 2030 and 783 million by 2045 globally. Cases of T2DM have been rising steadily and the numbers have doubled in the last 20 years, making it a significant public health challenge across the globe (Richardson, Zaletel, & Nolte, 2016). T2DM represents 90% of all diabetes cases and the trend is similar in the United Kingdom (UK) which currently has 4.7 million people living with diabetes, but the figure will have risen to >5.5 million by 2030 (Whicher, O'Neill, & Holt, 2020). WHO, 2021). Prolonged hyperglycaemia has potential to cause microvascular complications such as retinopathy (eye damage), nephropathy (kidney disease), neuropathy (nerve damage) leading to leg ulcers, infection, and amputation, and also macrovascular complications such as cardiovascular disease (e.g., myocardial infarction), (Chawla et al., 2016; ADA, 2022, WHO, 2021). Diabetes also caused 1.5 million deaths globally in 2019 (WHO, 2021). In addition, diabetes has an economic impact on the healthcare sector. According to IDF (2021) estimated costs at least $966 billion dollars in health expenditure - 9% total of spending on adults.

b. Management of T2DM

Self-management is an important element in T2DM since unhealthy lifestyle is directly implicated in most of the ensuing complications (Van Smoorenburg et al., 2019). It is defined as the active patient participation in the treatment of their conditions, and it has three basic set of activities. Medical management entails medication compliance and adherence to dietary requirement while behavioural management involves adopting new protective behaviours with regards to the chronic disease in question (Van Smoorenburg et al., 2019). The specific activities in diabetes self-management include adopting a healthy diet plan that excludes high fat foods, regular physical exercising, smoking cessation, reduced alcohol consumption, foot care and self-monitoring of blood glucose (SMBG) (Shrivastava, Shrivastava, & Ramasamy, 2013). Lastly, emotional management requires patients to develop adequate coping behaviours towards the feelings of frustration, despair and fright that are associated with chronic disease (Van Smoorenburg et al., 2019).

Self-management is critical in T2DM especially for patients under insulin therapy as they have limited contact (only three hours every year) with care providers (Whicher et al., 2020).

Thus, self-regulation of blood glucose through medication, behavioural and emotional management becomes their personal responsibility in the remaining 8757 hours and (Van Smoorenburg et al., 2019). Patients must be educated on self-management and training modules touch on multiple aspects relevant to diabetes. They include consistent blood glucose monitoring, medication, healthy eating, physical activity, problem solving and adequate coping with psychosocial issues (Anderson et al., 2021b; Powers et al., 2016). The modules are delivered by experts in clinical, psychosocial, behavioural and educational diabetes care for comprehensive coverage of all relevant concepts (Powers et al., 2016)

T2DM is a chronic and progressive medical condition that is predominantly self-managed by the individual (NICE, 2016). It involves lifestyle modifications, and treatment that can be challenging for individuals. One of the management strategies of T2DM is self-management via structured education programmes (Diabetes UK, 2022). Through a structured education programme, adults with T2DM can improve their knowledge and skills as well as motivate them to take control of the disease and self-manage it effectively. Thus, it is important for the emotional and psychological needs to be incorporated in the self-management education modules for patients with diabetes as recommended in diabetes related health policies.

c. Women with T2DM

With the steep rise of T2DM and its associated complications comes mounting evidence demonstrating clinically significant differences between men and women (Kautzky-Willer et al., 2016). A recent study by Stewart (2022) which was conducted in England 2020-2021, found that 55.7 % of those with T2DM were men, compared with 44.3 % of women. Older studies, however, have indicated a higher frequency of diabetes among women than among men based on age, education level, hypertension, and BMI (Duboz et al., 2012). Women with diabetes may also be at a greater risk of developing complications than men (Kautzky-Willer et al., 2016). According to Peters et al. (2015), diabetes increases the risk of cardiovascular complications by about four times in women compared to two times in men. In addition, women are more likely to suffer diabetes related complications such as vision loss, kidney disease and depression (Allen & Sesti 2018; WHO 2021). There are also there are emotional health deficits associated with living with T2DM.

Moreover, approximately 40% of individuals living with T2DM have poor psychological health (Whicher et al., 2020). Diabetes distress (DD) is the primary emotional challenge in women with T2DM. It arises from the pressure of having to undergo constant monitoring and

use medications to manage T2DM. The persistent worry over complications and impacts of diabetes on personal and professional life also contribute to DD (Kalra et al., 2018; Robinson, Luthra, & Vallis, 2013). Anxiety and depression are also reported in individuals with T2DM. Anxiety affects 14% of patients with diabetes while depression affects 30% and the latter magnifies the diabetes symptom burden. In the UK, one in five people with insulin-treated T2DM have DD whereas one in six people with non-insulin treated T2DM have DD (Diabetes UK, 2022). DD, anxiety, and depression should be distinguished in diabetes as they different psychological experiences and health outcomes. However, adverse outcomes are associated with DD more than the other psychological disorders (Kalra et al., 2018; Robinson et al., 2013).

d. Midlife women with T2DM

Women whose periods stopped between 40-44 years were 2.4 times more likely to develop diabetes whereas women between 45-55 years were 60 % more likely to develop the condition compared to menopause at later ages (Muka et al., 2017). Midlife runs from the late reproductive stage (35-40 years) to late post-menopause (<65 years) and presents several health issues for women (Harlow & Derby, 2015). It is also an important and potentially salient time for women to make health behaviour changes. Factors such as weight gain leading to being overweight (or obesity) and an increase in age are associated with oestrogen depletion (Lizcano and Guzmán, 2014). Age and increasing weight are also common risk factors for the development of T2DM (Public Health England, 2014). In addition, hormone changes are known to alter insulin sensitivity and glucose metabolism biologically (Wilcox, 2005). A case study found that an increased prevalence of insulin resistance was seen in the postmenopausal women as compared to the premenopausal women (CV, Balaji and Seethalakshmi, 2012). Midlife women have a higher prevalence rate of T2DM during menopause (Muka et al., 2017). Similarly, hormone changes have been shown to affect a woman's quality of sleep. Mainly because of night sweats and hot flashes caused by hormone changes, about 42% of premenopausal and 60% of postmenopausal women reportedly have sleep disturbances (Jehan et al., 2015). For those with diabetes, sleep difficulties could be worsened by various diabetes symptoms and related medications that can cause more frequent urination. Thus, there is a need for increased health care focus to support midlife women with T2DM.

d. Women Wellness Type 2 Diabetes Programme for Midlife Women

The Women's Wellness with Type 2 Diabetes Program (WWDP) is a multi-modal intervention, designed to be delivered through an eHealth website and hard copy targeting the

health needs of midlife women living with type 2 diabetes (Anderson et al., 2021a; 2021b). A prior international feasibility study of the WWDP evaluated the feasibility of participant recruitment and retention rates for the programme, as well as the program's initial efficacy in improving wellbeing outcomes in Australia and UK (Anderson et al., 2021a). According to the results of the quantitative and qualitative studies (Anderson et al, 2021a; 2021b), there was a need to adapt the results into WWDP before going to full trial. The WWDP has been newly adapted following an empirical and theoretical evidence review and engagement of Patient and Public involvement (PPI). The online newly designed WWDP includes new components which are goal settings (establishing individualised assessment of outcome) and peer support group (establishing engagement of participants) which have not been tested before, additionally it will be conducted only in the UK. This revision process with the PPI, along with the review evidence, demonstrated the need for a feasibility evaluation before proceeding with the full trial. In the next sections, WWDP 1 refers the previous version whereas WWDP+ refers new version. The details of the intervention will be explained under the section 21.

9. Aims of the Study

The aims of the study to evaluate a) the feasibility of the newly designed WWDP for midlife women in the UK and to explore the participants' experiences with the content and delivery of the new online intervention, b) the research protocol for evaluating the effectiveness of the WWDP on improving outcomes.

10. Research questions

Protocol feasibility:

1. Is participant recruitment, retention, and data completeness at 12 weeks comparable to the 12-week outcomes in the previous feasibility trial?
2. Can we recruit midlife women with a diverse range of ethnic backgrounds through online methods?
3. What are the barriers and opportunities to maintaining and retaining participants in the peer support group of WWDP?
4. Have any WWDP adaptions resulted in changes to the intervention effect signal compared to the original intervention?

Intervention feasibility:

1. Is the newly designed WWDP acceptable to women?

2. Will women continue to access the newly designed intervention?

3. What NHS healthcare do women access whilst undertaking the intervention?

4. Do midlife women engage with, and sustain involvement with the peer support component of WWDP?

11. Potential beneficiaries

Participants: Persons who participate in this study will help to assess whether WWDP+ fits the needs of midlife women with living T2DM. This study cannot guarantee that all participants will personally benefit from the research findings, but the evidence generated from this study begins to shape the design and delivery of the peer support component of the WWDP+ that has not been tested before.

12. Participant's inclusion and exclusion criteria

Recruitment for the Study 1 of participants will be based on the following criterion.

12a. Study 1

Inclusion Criteria

- Women aged ≥ 45 and ≤65 years
- A current diagnosis of T2DM
- DD score of 2 or above on the DDS scale (Polonsky et al., 2005) to ensure participant has diabetes distress before allocating to intervention.
- Using medication to treat T2DM (insulin or oral medication)
- Living in the UK and with access to diabetes care in the NHS
- Able to read and speak English
- Access to a computer or laptop, internet access in public or workplaces and IT literacy (defined as being able to buy a product over the internet, search for a topic and send an email).
- Have a Facebook account or will be able to accept to open a new Facebook account.
- Women receiving all diabetes care through their GP surgery.

Exclusion Criteria

- Women diagnosed with type 1 diabetes or GDM
- Unable to read and understand English
- Women without computer or internet access.

- Women who currently participate in other research that could be impactful to this study, such as interventional studies that could interfere with/impact this study.

13. Setting

An external digital advertising agency will be used for a social media recruitment strategy to reach and engage a diverse group of women with T2D between the ages of 45 to 65 in the UK. The agency specialises in health care related projects and advertise mainly through digital platforms such as Google, Facebook, Twitter and Instagram. A Microsoft form will be designed by IPN and will be accessible through these platforms for the women to complete, provide their demographic details i.e. age, ethnicity, location within the UK, indicate if they are interested to engage with research, PPI and/or peer support activities and provide their email address. The social media campaign will be advertised for 4 weeks through Facebook and Google. The advert consisted of a landing page promoting King's College London and its interest to recruit of women with type 2 diabetes, primarily to take part of PPI workshops and future research activities. The Microsoft form where data will be collected on the individual person interested to be contacted.

Participant recruitment

The following steps will be taken to identify the eligible participants.

Step 1. Women who express interest in participating research will be contacted via email. The PI will send an email about https://www.kcl.ac.uk/research/wwdp that includes Participant information sheet, eligibility criteria. If potential participant feels matching with criteria and consider participating, then will follow the instructions to fill the screening questionnaire including diabetes distress scale (DDS) through Qualtrics (https://qualtrics.kcl.ac.uk) for high distress for eligibility (Polonsky et al., 2005). A minimum of 24 hours will be offered to women to think and decide if they are interested in participating after filling out screening questionnaire. The PI will send one reminder about participating.

Step 2. Once participant eligibility is confirmed, Informed Consent will be undertaken. Participant Information Sheets (PIS) and consent forms will be shared with participants via Qualtrics (https://qualtrics.kcl.ac.uk). They will be asked to give an informed written consent form as online. Also, a copy of signed informed consent form will be sent to the participant's email address.

14. Research Design

The study has been designed following established two phases for assessing feasibility of the newly designed intervention.

14a: Feasibility Study (Study 1): This phase, hereafter refer to as 'Study 1' is a nonrandomised single arm study (n=40) to aid refinement of the intervention for the next phase of the study. In this study 1, forty eligible women will receive the WWDP+.

The specific objectives are as follows:

1. Will we be able to recruit 50% of women that express interest into the study?
2. Will pre-specified progression criteria be met in the time frame?
3. Will midlife women set up their goals?
4. How many goals midlife women will set up for 12 weeks?
5. Will midlife women meet their goals at the end of 12 weeks?

14b. Sample Size for Study 1: A sample size justification is important for pilot studies; however, traditional power calculations are not appropriate for feasibility studies' sample size calculation (Arain et al., 2010; Lancaster et al., 2004; Whitehead et al., 2016). The purpose of feasibility trials is not to establish the superiority of an intervention, but to test procedures and processes and to estimate some of the parameters for the definitive trial. (Arain et al., 2010). Julious (2005) recommends that the minimum sample size be 12 participants per treatment arm, whereas Teare et al. (2015) recommend a total of 70 participants in order to reduce the level of uncertainty around the estimation of the standard deviation. Considering that this is a feasibility trial, this study will recruit 40 participants.

14b. Outcomes and outcome measures

To address research questions the following data will be collected. The below outcome measures were chosen in the WWDP 1 and performed well in relation to the completeness of data and effect signal. Thus, they will be used in the WWDP+.

- Socio-Demographic & Biomedical Data: Age, marriage status, educational level, duration of diabetes, treatment method, house income and employment status. Biomedical data (medication usage, health history and immediate family health history, diabetes diagnosis date and treatment methods) will be collected. HbA1c will be also collected as self-reported by participants.

- Feasibility outcomes: Data based on the PI records will be collected on the number of persons who registered interest, eligible participants who were recruited (i.e. provided consent to participate in the study), allocate to intervention, participants who completed the intervention period and those who completed all assessments will be captured. In order to determine feasibility, the following cut-off points (pre-specificity progression criteria) will be used: 50% recruitment of all eligible participants, and 70% completion rate (including all assessments, interventions, and follow-ups).
- Diabetes Distress: The Diabetes Distress Scale (DDS) will be used once to screen before baseline for recruiting eligibility of high DD and to assess at baseline and post intervention follow up (Polonsky et al., 2005). The instrument contains 17-items categorized into four subscales labelled as Emotional Burden (five items), Regimen Distress (five items), Interpersonal Distress (three items) and Physician Distress (four items). The DDS has a good internal consistency (Cronbach's $\alpha = 0.95$), and all subscales have a Cronbach's alpha > 0.87). This instrument measures distress that has a consistent, generalizable factor structure and good internal reliability and validity across four different clinical sites. Previous study has successfully used the DD scale to assess distress among midlife women with T2DM (Anderson et al., 2021a).
- Self-Efficacy: The Diabetes Management Self-Efficacy Scale (DMSES UK) assesses self-efficacy for T2DM management (Sturt, Hearnshaw & Wakelin, 2010). It is a 15-item scale for T2DM self-management actions and behaviours. Sturt, Hearnshaw and Wakelin (2010) conducted a study and found that the scale has good internal reliability, internal consistency, construct validity, criterion validity, and test-retest reliability.
- Quality of Life: The Short-Form 36 (SF–36) assessed health-related QOL across 9 domains: physical functioning, physical health role limitations, emotional problems, role limitations, pain, mental health, social functioning, pain, general health, reported health transition and overall, mental-and physical well-being scores (Ware, 1992).
- Physical Activity: The International Physical Activity Questionnaire (IPAQ) assesses physical activity (Craig et al., 2003). This 9-item as short form self-reported measure of physical activity for use with individual adult patients aged 15 to 69 years old. The IPAQ can be used clinically and in population research that compares physical activity levels between populations internationally.
- Sleep: The General Sleep Disturbance Scale assesses participants' sleep (Lee, 1992). This 21-item scale includes subscales for problems initiating sleep, waking up during

  sleep, waking too early from sleep, quality of sleep, quantity of sleep, fatigue and alertness at work, and use of substances to induce sleep.

  - Physiological Measures: Anthropometry measures will be included height and weight body mass index (BMI), waist and hip circumferences. BMI will be grouped according to the WHO International Classification of adult weight (i.e., <18.5 underweight, 18.5–24.9 in normal weight range, and 30 obese) (WHO, 2000). These measures will be self-reported.
  - Menopause management: The standard Greene Climacteric Scale will be measured menopausal symptoms, with 21 items assessing subscales for vasomotor, somatic, psychological (anxiety and depression), and sexual function symptoms (Greene, 1990;1998).
  - Habitual Dietary Intake: the UK Diabetes and Diet Questionnaire (UKDDQ), asks about consumption of higher energy foods and drinks, fruit, vegetables, oily fish, common higher fibre cereal foods and meal patterns to indicate the usual frequency of consumption over the period queried. It takes roughly 10 minutes to complete and can be self-scored (England et al., 2017).
  - Intervention Use Data: Usage of the peer group refers to how many times midlife women have logged on, what proportion of time women spent for 12 weeks.
- Goal settings: Goal Attainment Scale (GAS) (Kiresuk and Sherman, 1968). GAS gives individuals the ability to construct their own outcome measures; this contrasts with measures that are based on a standard set of tasks. It encourages patient involvement. According to Turner-Stokes (2009), rehabilitation goals are more likely to be achieved when patients and their families are involved. Furthermore, introducing formalised goal setting leads to more information sharing at the beginning of a rehabilitation process.
- Smoking Measurement: Selected smoking prevalence questions will be measured as recommended by a subset of key questions from the Global Adult Tobacco Survey.

14c. Data collection

For Study 1: PRO Data will be collected at baseline and then follow-up data will be collected at 3-months and 6-months post-baseline. The baseline and follow up assessment data will be undertaken by the principal investigator through an emailed survey using Qualtrics software (https://qualtrics.kcl.ac.uk). Advantages of this method include convenience and better responses, and accessibility from any device. Moreover, due to the geographical diversity

of the UK, the use of face-to-face rather than online data collection might exclude participants who live far away from London.

Data for the surveys will be collected via Qualtrics, which is a university supported GDPR compliant secure survey platform, using a Qualtrics/ King's College account. Following participant consent to allocate intervention, use of the peer group by participants will also be automatically recorded in order to provide numbered counts of logins and time spent on the Facebook group from first day of the intervention to end of the twelfth weeks.

The newly designed WWDP website will include a video with instructions on how to perform waist and hip circumference measurements that has been created by Diabetes UK (2021). This is necessary, as participants will self-report these data using the biomedical data sheets.

Participants will be also asked to complete a duplicate form of their goal setting/evaluation form and return it by email to the researcher within seven days of receiving the form at 3 weeks and at 12 weeks. Each goal-setting sheet will be given a unique identifier so that goal-setting sheets could be linked to participants questionnaires. Participants will be asked if they have identified a goal, decided on an action-plan and if they have started it. Participants will asked if they have decided on an action-plan on completion of WWDP, what their action-plan is, if they are still working on their action-plan, if they have discussed their action-plan with others, and perceived success in achieving their action-plan. Perceived success is assessed using the following five response items: "Much better than expected / Somewhat better than expected / Expected level of attainment / Somewhat less than expected / Much less than expected". Respondents will be also asked if they have set any new action-plans following completion of WWDP, what these action-plans will and perceived success at meeting any new action-plan. Below table 1 demonstrates a summary of data collection timeframe.

Table 1: Timeframe of data collection

DATA	Pre-intervention	At Baseline	Goal Setting at 3 Weeks	3 and 6 Months post baseline Follow up
Eligibility screening question	✓			

include age, sex, T2DM diagnosis and Diabetes Distress Scale (inclusion criteria)				
Socio-Demographic		✓		
Biomedical Data		✓		✓
Feasibility data		✓		✓
DDS for outcome		✓		✓
DMSES UK		✓		✓
The Short-Form 36 (SF–36)		✓		✓
IPAQ		✓		✓
The General Sleep Disturbance Scale		✓		✓
Anthropometry measures		✓		✓
The standard Greene Climacteric Scale		✓		✓
FFQ		✓		✓
Intervention Use Data				✓
Goal Attainment Scale			✓	✓

14d. Data Analysis

For Study 1: The recorded peer group data will be examined to identify the average number of logins for participants and their spending time. Adherence to the intervention will also be examined in terms of the number of users and nonusers of the group at each time point to identify adherence over time and by population group. Descriptive statistics will be used to characterize rates of demographic characteristics, recruitment, retention, and completed data. For continuous data, means and standard deviations will be calculated, as well as frequency and percentages for categorical variables. Quantitative data will be analysed using the Statistical Package for Social Sciences version 29.0 for Windows.

15. Research Design for Study 2

15a. Participants and Setting for Study 2

Inclusion Criteria:

- All inclusion criteria from the feasibility trial will be included.
- Participants who log into the online peer group on at least three occasions over the twelve weeks.

Exclusion Criteria:

- All exclusion criteria from the feasibility trial.
- Participants who do not attend the peer support group during the intervention.

Participants will be recruited in order to above eligibility criteria for Study 2. Participants will be informed in the consent form about recruitment after the completion of the intervention duration.

Public & Patient Involvement (PPI): This protocol has been reviewed by two members of our PPI group who represents women from the target population. This group gave the feedback about other ethical documents (e.g. consent form, interview topic guide) to ensure that all documents are understandable. The PPI group will be consulted later to discuss questions in the interview guide potential issues when conducting the interviews.

15b. Semi-Structured Qualitative Interviews (Study 2): This study hereafter called 'Study 2' will be a qualitative study (n=20) will be conducted after completion of Study 1. The aim of the involving individual semi-structured in-depth interviews is to understand how the newly designed WWDP was used and what its impacts were.

The specific objectives are as follows:

1. To explore the participants' experiences with the content and delivery of the newly designed intervention.
2. To examine participants' perspectives on the barriers and facilitators of peer support.
3. To explore participants' experiences of the process related to goal attainment.

15c. Sampling: According to Curtis et al. (2000), purposive sampling consists of developing the sampling plan based on the study objectives and the research questions. Hence, the study will benefit from purposive sampling since it is pragmatically driven by the research questions and study objectives (Miles & Huberman, 1994). As a result, a maximum variation sampling approach will be used. As a first step in constructing a sampling frame, key dimensions of variation are identified with regard to age, ethnicity, duration of diabetes, baseline DD scale, engagement of specific elements (progression criteria) of WWDP+. A sampling frame (Table 2) demonstrates as a list including all the items regarding women who consent to share views about WWDP+.

Table 2: *Sampling Frame*

Variable	Aim	Rationale
Age ranges	Recruit >3 participants per age band as below<br>45-50<br>50-55<br>55-60<br>60-65	Ensuring the included all age groups in this range.
Ethnicity	Recruit >3 participants per ethnicity band as below<br>White<br>Black<br>Asian<br>Mix ethnic background	Understanding of a diverse range of ethnic backgrounds in terms of cultural diversity.
Duration of Diabetes	Recruit >3 participants per diabetes duration band as below<br><2 years	Assessing the full spectrum of needs of women who represent different stages of diabetes.

	2-5 years<br>6-10 years<br>>10 years	
Baseline DD	Recruit >3 participants per baseline DD<br>2-2.5<br>2.5-3<br>3>	Understanding the needs of women who have different levels of DD.
Engagement of specific elements of WWDP+	Recruit >3 participants per engagement during 12 weeks<br>a. Attendance of peer group<br>≥3 times<br>≥6 times<br>≥9 times	Understanding the engagement levels of WWDP+ on women.

15d. Sample Size for Study 2: In order to achieve data saturation, it has been recommended that qualitative studies require a minimum sample size of 12 (Braun & Clarke, 2013; Fugard & Potts, 2015; Guest, Bunce, & Johnson, 2006). Baker and Edwards (2012) also argued that the sample size for the qualitative studies may be determined based on the objectives of the study and the availability of resources. Since the study aims for 40 participants in the intervention group, based on the resources, a sample of 20 women will be recruited from the feasibility trial. The sample size may be revised in response to data quality as the study progresses.

15e. Data collection for study 2**:** One-to-one interviews will be conducted via Teams at sixteen weeks post-intervention following 3 months post baseline data collection (before final following up at 6 months post baseline). A topic guide has been developed based on research question to explore participants' experiences regarding the content and delivery of the intervention and the peer support component and any challenges encountered during discussions, and general prompts will also be used. The topic guide has been reviewed by PPI (Please see Appendix 2). During the semi-structured interviews, the same topics will be asked of all participants within a flexible framework, and participants will be encouraged to narrate

their experiences in their own words. To make participants feel comfortable, the questions will be general yet open-ended. The interviewing process will take 30 to 45 minutes or 45 to 60 minutes; however, all participants will be informed that they can end the interview at any time. Informed written consent via Qualtrics will be obtained to audio-record all interviews on an encrypted digital recorder to allow accurate transcription. Also, peer group discussions will be copied and saved into word doc from the chat on the KCL laptop.

15f. Data Analysis for Study 2: The discussion/chats on the peer support group will be analysed as data. In addition, all interviews will be transcribed verbatim as audio recordings. Qualitative data (peer group chats and interviews) will then be imported into NVivo 12 software (QSR International) and analysed using a pragmatic thematic analysis approach (Braun & Clarke, 2006). Thematic analysis is a process for identifying, analysing and reporting patterns within data. In addition to its flexibility, it can be used to generate a rich, detailed account of a certain aspect or point of view from a range of theoretical perspectives. The transcripts and coding will be managed through NVivo, as will the data analysis for the final report. Braun and Clarke's theoretical position will be the framework for constructing a thematic analysis of the interviews. As described by Braun and Clarke, thematic analysis involves six stages, including reading the text, generating initial codes, collating codes into possible themes, reviewing themes, clearly defining, and naming themes, and writing up the final analysis. Throughout the final analysis, verbatim quotations will be used in order to answer the specific objectives of the study. This will allow the researcher to examine the experiences of different participants, generating in-depth insights as well as highlighting similarities and differences (Braun & Clarke, 2006).

16. Data Management and Monitoring for Study 1&2

A unique identification number (ID) will be assigned to each participant and will appear on any questionnaire or document used to collect data. In order to input and store the collected data, an electronic database will be created using Microsoft Access Software. The data collected through the questionnaires and other source documents also peer support group messages (saved in word doc file) will be entered into the database, which will be securely handled by the principal investigator on a password protected KCL computer. In order to minimise the number of missing data, it will be necessary to maintain contact with participants throughout the study period. The principal investigator will design the survey with mandatory fields to avoid missing information. After data collection, the data as blinded with given ID will be 'transported' to SPSS software for processing and analysis.

17.Participants withdrawal from the study

Participants will be informed in the participant information sheet that after they provide consent, they can withdraw at any time if they want. However, participants will not be obliged to provide reasons for withdrawal if they do not want to. Participants may no longer be able to withdraw their data from the study after the analysis of the data because it would have been anonymised and analysed. This final withdrawal date for participants in Study 1 and Study 2 is up to 10 days after participants have submitted the survey or been interviewed by contacting the research team. Participants who wish to withdraw from the study and request that their data be withdrawn will be able to reach the principal investigator through the email provided at the bottom of the information sheet.

18.Potential Risks, Confidentiality and Anonymity

Physical Risk: The use of the intervention is associated with potential risks. It is important to be aware of any possibilities for injury during exercise, especially if the participant does not exercise regularly. To minimise the risk of injury from exercise, intervention programs are tailored to the individual. By allowing participants to gradually increase their exercise levels over time, they do not overexert themselves at the beginning. To prevent stiffness and promote flexibility, participants are also encouraged to stretch regularly. Due to the participant's involvement in reading the eBook every day, there may be a risk of tiredness on their eyes, and stiffness on their shoulders. During the program, the participants will incorporate some exercise into their daily routine, which could potentially reduce this risk.

Psychological risks: There may be some topics in the book that participants find uncomfortable or during engagement of peer group. However, if this should happen, participants will be informed in the participant information sheet that they may choose not to speak about topics or questions that make them uncomfortable. If they remain upset, they should talk to their usual diabetes provider to get a counselling support.

Other risks: Study 2 involves midlife women with T2DM, and interview topics cover general, health and diabetes questions. These is no evidence to suggest that participating will be upsetting or distressing. However, if this should happen, participants will be informed in the participant information sheet that they may choose not to speak about topics or questions that make them uncomfortable or that they can stop participating at any time without giving a reason. If they remain upset, they should talk to their usual diabetes provider to get a counselling support.

Research Safety Protocol

In this study, participants will be informed of what taking part in the research would involve and provided with a participant information sheet (please see Appendix 3). The participants will be given time to read the information sheet and if they have any concerns, they wish to discuss with the PI about participating in the research. They will find the PI contact details on this sheet if they would like to. Due to the nature of the intervention, the above risks might occur, thus, to ensure participants remain as safe as possible following their participation, all participants will be required to provide their contact details as their eligibility is confirmed. This will help the research team to make contact with them and say to them whether they may need further help and encourage them to contact their healthcare provider if any above risks occur during research. In this case, we will recommend contacting their GP or diabetes nurse because this could affect their T2DM and general health.

The diabetes-related mental health experience and outcome we assess and explore in the study is diabetes distress only. We are not collecting any other mental health outcome or experience data. The literature has indicated the diabetes distress assessment scale cut-off score of 2 or over (on a 1-5 scale) as a score that indicates diabetes distress will be having a negative impact on the participants self-care activities that can result in raised blood glucose levels, weight gain and diabetes complications. The higher the score the more at risk of these physical health problems they are. This is the purpose of the intervention and its evaluation. The intervention addresses these physical health risks and there is signposting throughout for people to contact their diabetes care professional if they have concerns.

Whilst the current evidence base does indicate a symptom overlap between depressive symptoms and diabetes distress (but not between diabetes distress and Major Depressive Disorder), the current evidence base does not extend to understanding diabetes distress cut-off score regarding someone being a risk in relation to their mental health. In the absence of evidence related to this we propose the following mitigations:

1) All baseline DD scores will be assessed by a member of the research team within 48 hours of receipt of the completed survey. If the baseline score is 3.5 or over the researcher will contact the participant to communicate their concern for the impact of their diabetes on their mental health and signpost them to resources available to them in case, they feel in need of mental health care.

Twelve week follow up diabetes distress data will also be reviewed within 48hrs of receipt and the baseline process also followed. At the beginning of the survey, also will be signposted the following statement: 'If you have been upset by any of these questions due to questions on the survey, please discuss with your healthcare provider as soon as possible'. Furthermore, in the information sheet, the additional details of support sources are included about immediate or non-immediate support for participants. Please see below chart for summary of safety procedures:

Management of Distressed Participant during Intervention in Need of Immediate and non-immediate support:

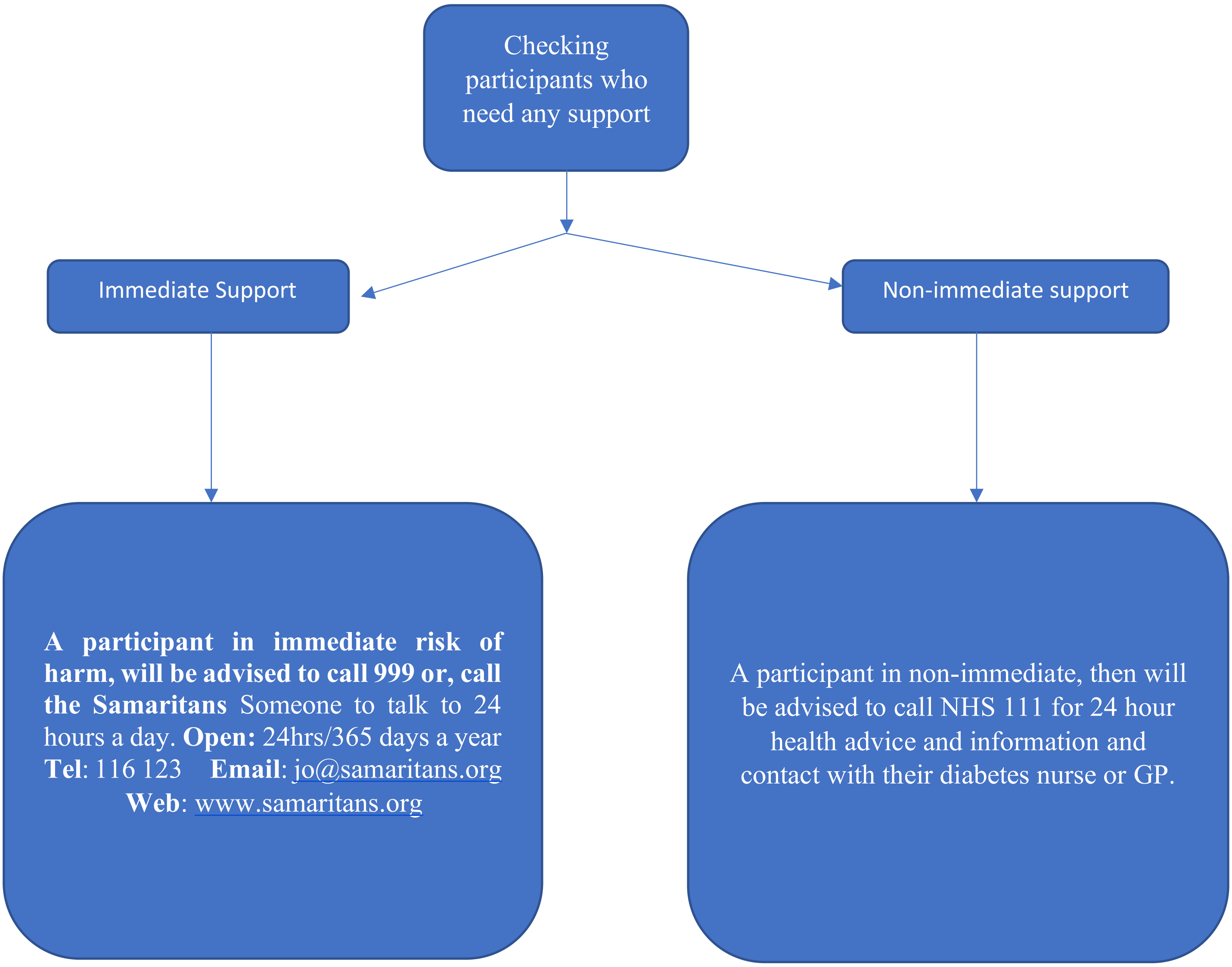

UK data protection law (including General Data Protection Regulation (GDPR)). We will maintain privacy and confidentiality by using Qualtrics which is a university approved survey tool. King's College London (KCL) is the sponsor for this study based in the United Kingdom.

The researcher will be using information from the participants in order to undertake this study and will act as the data controller for this study. This means that the researcher is responsible for looking after participant information and using it properly. Data will be handled in accordance with the General Data Protection Regulations (Information Commissioner Office (ICO), 2022). KCL will keep identifiable information about participants for 5 years after the study has the study has completed and afterward data will be destroyed permanently.

Pseudonymised data capable of being linked to a participant through a unique code number will always be used. This will be explained to the participants during the consenting process. The database storing participants personal data (names and contact information) will be separated from participants' research data. Each participant will be assigned an ID number such that no personal information is used to identify datafiles (including recordings). The database linking names and ID numbers will be stored separately to the main datasets and will be encrypted and held securely. Data on ethnic origin, health data and dates of birth will be tagged with the study ID number. Again, the database linking names and ID numbers will be stored separately to these datasets and will be encrypted and held securely electronically on KCL network. Audio-recordings will be stored electronically on KCL network and will be encrypted, and password protected. The answered questionnaires will be anonymised.

19. Ethical Consideration

Ethical approval for the study will be obtained from King's College Research Ethics Committee. Confidentiality and data protection for all participants will be ensured as discussed earlier under section 17. Due to nature of peer support component, to minimise potential harm, group rules and guidelines will be shared with participants. Please see separated attached file.

20. Dissemination

Two mains peer reviewed papers are expected to be developed from this study for publication in Open Access Journals. These will be presenting the study, methodology chapter and feasibility trial. We will aim to publish this in high impact factor. The International Diabetes Federation (IDF) and Diabetes UK Professional congress will be targeted for conference presentations and discussion of our project processes and outcomes.

21. Intervention Details

The previous version of WWDP was designed to assist midlife women with T2DM to effectively manage their condition and developed from the Diabetes Manual for South Asians

(DOSA) and the Women's Wellness programme (WWP) (Sturt et al., 2008; Anderson et al., 2015). The theoretical foundation of WWDP is Bandura's Social Cognitive Theory (SCT) whose emphasis is on enhancing self-efficacy as a strategy for initiating and maintaining health promoting behaviours. Self-efficacy refers to the belief in oneself to perform specific tasks and overcome the associated problems (Anderson et al., 2021b). Thus, the program combines self-directed tools including interactive books, websites, and direct consultations with health professionals via videoconferencing to support the development of self-efficacy (Anderson et al., 2021b, 2021a). In the next section, the components of intervention will be presented.

- Website and eBook Component of WWDP

The newly designed intervention is a 'Women's Wellness Type 2 Diabetes Programme', which is a 12-week multi-modal intervention through an eHealth website. The website has a specific terms and conditions and participants will be asked to read this information when log into website (Please see Appendix 4). It aims to reduce diabetes complications and promote healthy behaviours and is theoretically underpinned by Social Cognitive Theory (Bandura, 1997), with an emphasis on perceived control and planned behaviour as concepts that promote health behaviour change. It includes a web interface (including podcasts), and an interactive electronic book (eBook which is accessible from any device) that offered detailed intervention instructions and guides for participants to record relevant health and lifestyle information based on UK and includes peer support on Facebook private group align with peer supporters (Participants will be able to create new discussion threads and comment on others, also they can interact with another. This will be explained in the next section).

The eBook encourages women to bring together the health topics in 4 stages and incorporate them into their lives over a 12-week period. Chronologically, the 4 stages include: 1) preparation and changing lifestyle (including diabetes specific refresher); 2) establishing healthy lifestyle habits; 3) maintaining health for illness prevention; and 4) becoming independent. Included activities allow participants to record responses and reflections, including a weekly exercise planner and a weekly health behaviour review exercise. Please see below table 3 for the intervention contents.

Table 3: *Participants will read the Components from the Women's Wellness with Type 2 Diabetes Programme Book every day. The sections to read are scheduled on daily bases from week 1 to week 3. From week 1, we expect participants to be able to follow topic by themselves according to their chosen goal. Therefore, the sections to read are only scheduled on a weekly basis and on different topics.*

**Step 1**		Day 1	A refresher about type 2 diabetes

**PREPARATION & CHANGING LIFESTYLE**	**Week 1 – Preparation**	Day 2	Your feelings about diabetes
		Day 3	Coming to terms with your diabetes<br>Treatment and what it means for you<br>Illness – what to do when you feel unwell
		Day 4	Starting to think about routines and balance in your life<br>Your self-care plan
		Day 5	Overcoming obstacles to change
		Day 6	Your goals for health
		Day 7	Reflection
	**Week 2- Changing Lifestyle**	Day 1	Healthy eating with type 2 diabetes<br>Water
		Day 2	Understanding more about glucose
		Day 3	Alcohol and smoking
		Day 4	Healthy shopping and meal planning
		Day 5	Regular physical activity and exercise
		Day 6	Stretching and flexibility<br>Balance<br>Strengthening the pelvic floor
		Day 7	Preparing for the week ahead<br>Week 3 exercise schedule
	**Week 3 – Changing Lifestyle**	Day 1	Changing your eating behaviours
		Day 2	Strength training exercise<br>Program 1: Exercises without equipment
		Day 3	Stress and you
		Day 4	Diabetes distress<br>Strategies for managing stress
		Day 5	Better sleep<br>Memory and thinking
		Day 6	Goal setting
		Day 7	Review<br>Review of week 3<br>Week 4 exercise schedule
**STEP 2: ESTABLISHING HEALTHY**	**Week 4- Healthy weight**		Healthy weight week<br>Review of week 4<br>Week 5 exercise schedule

**LIFESTYLE HABITS**	**Week 5- Strong Bones**		Strong bones week<br>Osteoporosis prevention<br>Getting enough calcium and vitamin D<br>Program 2: Exercises with dumbbells or resistance bands<br>Program 3: Exercises in a fitness centre<br>Review of week 5<br>Week 6 exercise schedule
	**Week 6- menopause**		Let's talk about menopause<br>Expectations<br>Menopausal symptoms checklist<br>Managing menopausal symptoms<br>Sexuality and menopause<br>Let's review the steps you have taken<br>Review of week 6<br>Week 7 exercise schedule
**STEP 3: MAINTAINING HEALTH FOR ILLNESS PREVENTION**	**Week 7 – Healthy Heart**		Healthy heart week<br>Review of week 7<br>Week 8 exercise schedule
	**Week 8- Diabetes Complications Prevention**		Diabetes complications prevention week<br>Your regular tests and check-ups<br>Preventing complications of diabetes<br>Short- and long-term complications<br>Review of week 8<br>Week 9 exercise schedule
	**Week 9 – Cancer Prevention**		Cancer prevention week<br>Goal setting<br>Review of week 9<br>Week 10 exercise schedule
**STEP 4: BECOMING INDEPENDENT: REVIEW, MOTIVATION AND LOOKING FORWARD**	**Week 10 - Review**		Reviewing what you have learned<br>Review of week 10<br>Week 11 exercise schedule
	**Week 11- Motivation and change**		Motivation and change<br>Overcoming obstacles to change<br>Review of week 11<br>Week 12 exercise schedule
	**Week 12 – Goal Setting**		Looking forward – goal setting<br>Review of week 12<br>Final word

Fact Sheets also provide further information on different health related topics. There are also refillable sheets that allow to record and review the behaviours undertaken of the weekly activities at the end of each week in a separate online logbook. All participants will receive health education materials which are 1) an interactive website (Appendix 6) which includes an

electronic book (eBook), fact sheets and logbook, 2) accessing to an interactive online peer support group on Facebook, 3) Personal Goal evaluation form (Appendix 5).

Table 4 below describes the intervention's focus the targeted knowledge and behaviours included. All these activities that women are being asked to do will lead (or not) to improved outcomes, and these all directly relate to self-efficacy theory, as shown in Appendix 7. All participants will continue to receive their usual care, including care from their usual general practitioner, diabetes nurse and any other health information normally provided by other health care providers.

Table 4*: Targeted health knowledge and behaviours (adapted from Anderson et al., 2021a)*

Knowledge / Behaviour	Recommendations
1. Stress and psychological wellbeing	• Sleep<br>• Develop healthy stress management strategies<br>• Reduce anxiety and depression<br>• Manage Diabetes Distress
2. Diabetes self-management	• Medication concordance<br>• Blood glucose management<br>• Managing clinical appointments
3. Physical activity	• Be moderately physically active, equivalent to brisk walking for at least 30 minutes per day. As fitness improves, aim for 150-300 minutes of moderate intensity exercise per week<br>or<br>75 to 150 minutes of vigorous intensity physical activity per week<br>• Complete strength exercises on two or more days a week that work all the major muscles.
4. Diet	• Consume at least 5 x 80g portions of a combination of fruit and vegetables per day. This is five portions of fruit and vegetables, not five portions of each (UK National Health Service (NHS), 2018).<br>• Eat mostly foods of plant-based origin<br>• Limit consumption of energy-dense foods

	• Avoid sugary drinks and snacks<br>• Limit intake of red meat<br>• Manage portion size of meals<br>• Consumption of recommended alcohol intake per country guidelines (NHS, 2017)
5. Body fatness	• Be as lean as possible within the normal weight range<br>• Avoid weight gain and increases in waist circumference
6. Smoking	• Smoking cessation
7. Menopausal symptoms	• Management of menopausal symptoms
8.Preventative health and risk screening	• Heart disease, eye health, renal health, breast, and gynaecological health

- Peer Support Component of WWDP

Peer support group as online offer the opportunity to meet other people who are living with T2DM, to share experiences and information, learn more about diabetes and living with a lifelong condition, support each other, discover support and activities on Facebook. Prescott et al., (2020) conducted a study related to peer support group on Facebook in the UK and US. The study highlights the positive impact of shared personal experiences and offers a greater understanding of the benefits of online peer to peer support for mental health and wellbeing (Prescott et al., 2020). Participants will be expected to log into the online peer group on three occasions of the twelve weeks. Peer supporters will moderate the peer group five days a week during intervention duration. They are non-professionals who have T2DM. The peer supporters' main tasks will the following: organisation of online group meetings, guiding about group rules and exercise units as well as facilitation of group discussion on diabetes related topics, support of physically weak group members and motivation of unmotivated participants. Throughout all responsibilities, peer supporters will encourage to rather give support than advice. They will be trained following topics: the concept of peer support, organisation of group meetings, physical activity, motivation, nutrition, experience, and feedback.

The user guideline and ground rules (Appendix 8) will be able to read on the intervention website by participants for peer support group also will be accessible by participants and will be shared by peer supporters ahead of the intervention. When the woman is introduced to the

intervention, then participants will be allocated to peer support group. Management details of peer support group has been included user guideline (Appendix 8).

- Goal Setting Component of WWDP

When the woman is introduced to the intervention, the PI will share a personal action-plan on Personal Goal Evaluation form with GAS which has a unique identification number with participants via email (Appendix 5). Participants will be encouraged to read week 1 on the eBook and set up their goals within peer supporters' motivation through peer group. This will include goal-setting session as the culmination of the programme where the peer supporters discuss the benefits of making an action-plan guide peer group to identify areas to target. By the end of the session of Week 1, participants will have the opportunity to identify their own personal goals and write it the form. Participants will be encouraged to set "SMART" (i.e., specific, measurable, attainable, realistic, and timely) goals and to focus preferably on one or two goals. As part of the process, participants will be encouraged to think potential barriers to their action-plans and potential solutions and to rate their confidence (score to 1-10) in attaining their chosen goal on the GAS evaluation form by peer supporters. Those with a self-assessed confidence level of less than 7 are encouraged to re-visit their goal or think of ways to increase their confidence by peer supporters.

- Podcast component of WWDP

The intervention also includes podcast section. This will help women to hear evidence-based information through short talking by Diabetes UK resources link. The podcast topics include below contents:

1. Why women need to talk about type 2 diabetes by Prof Jackie Sturt
2. Treatments
3. Foot Checks
4. Sleep
5. Stigma
6. Mental Wellbeing

- Delivery of Intervention

Once baseline data collection is completed, participants will be enrolled onto the programme with the researcher as described in. The newly WWDP delivery in online and self-directed. The PI will enrol in Table 5.

Table 5: *Steps that participants will follow to access the intervention.*

Step Number	Step Details
1	Emails will be sent to participants and peer supporters with a link to website details and log-in instructions.
2	Participants and peer supporters will create their anonym username account on the website.
3	Website homepage will contain a welcome message from the PI with detailed steps for intervention.
4	Peer support guidelines and ground rules (also with peer supporters) will be accessible by website and Personal Goal evaluation form will be shared via email.
5	A peer support group, where participants can choose to be anonymous, with moderators (the principal investigator and three peer supporters) will allow participants to share their progress experiences align with guidelines and ground rules. (Guidelines and group rules have been attached in different file Appendix 8)
6	Regular reminders will be sent via peer support group for weekly log-in by peer supporters and automatically from website weekly (Pirolli et al., 2017)
7	Participants can contact the PI via email at any time.

22. Figure 2: Consort diagram for participants in feasibility trial

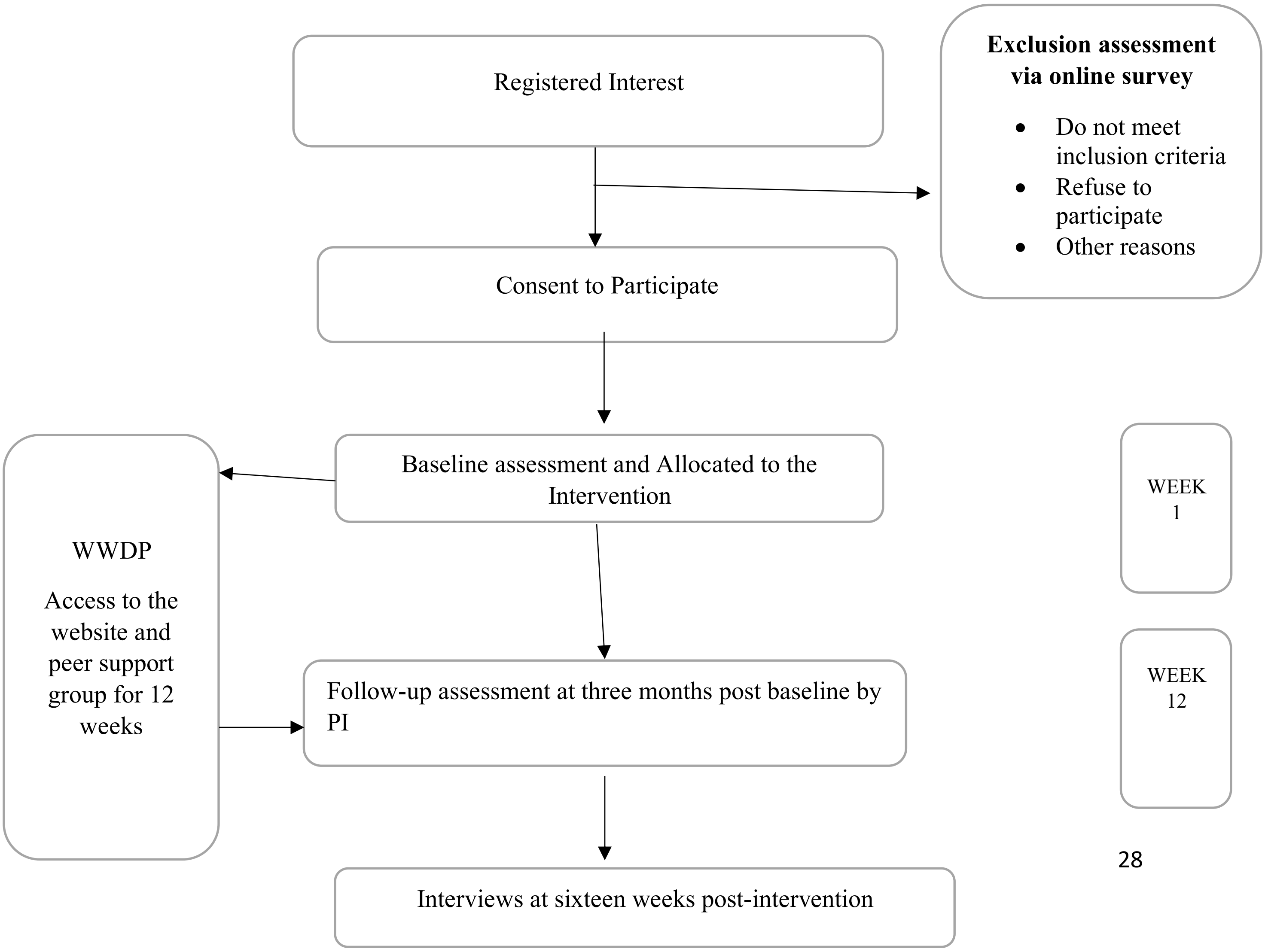

REFERENCES

Allen, J., & Sesti, F. (2018). *Health inequalities and women-addressing unmet needs*. London, England. Retrieved from https://www.bma.org.uk/media/2116/bma-womens-health-inequalities-report-aug-2018.pdf

Anderson, D., Seib, C., McGuire, A., & Porter-Steele, J. (2015). Decreasing menopausal symptoms in women undertaking a web-based multi-modal lifestyle intervention: The Women's Wellness Program. *MATURITAS, 81*(1), 69–75.

Anderson, D., Sturt, J., McDonald, N., Sapkota, D., Porter-Steele, J., Rogers, R., Temple, A., Seib, C., McGuire, A., Tjondronegoro, D., Walker, R., Al-Khudairy, L., & White, C. (2021b). Women's Wellness with Type 2 Diabetes Program (WWDP): Qualitative findings from the UK and Australian feasibility study. *Diabetes Research and Clinical Practice*, *172*, 108654. https://doi.org/10.1016/j.diabres.2021.108654

Anderson, D., Sturt, J., McDonald, N., White, C., Porter-Steele, J., Rogers, R., Temple, A., Seib, C., McGuire, A., Tjondronegoro, D., Walker, R., & Sapkota, D. (2021a). International feasibility study for the Women's Wellness with Type 2 Diabetes Programme (WWDP): An eHealth enabled 12-week intervention programme for midlife women with type 2 diabetes. *Diabetes Research and Clinical Practice, 171*, 108541–108541. https://doi.org/10.1016/j.diabres.2020.108541

American Diabetes Association (2022). Introduction: Standards of Medical Care in Diabetes-2022. *Diabetes care*, *45*(Suppl 1), S1–S2. https://doi.org/10.2337/dc22-Sint

Arain, M., Campbell, M. J., Cooper, C. L., & Lancaster, G. A. (2010). What is a pilot or feasibility study? A review of current practice and editorial policy. *BMC Medical Research Methodology*, *10*, 67. https://doi.org/10.1186/1471-2288-10-67

Baker, S. E., Edwards, R., & Doidge, M. (2012). *How many qualitative interviews is enough? Expert voices and early career reflections on sampling and cases in qualitative research*. National Centre for Research Methods, Southampton.

Bandura, A. (1977). Self-efficacy: Toward a unifying theory of behavioral change. *Psychology Review*, *84*, 191-215.

Braun, V., & Clarke, V. (2006). Using thematic analysis in psychology. *Qualitative Research in Psychology,* 3(2), 77–101. https://doi.org/10.1191/1478088706QP063OA

Braun, & Clarke, V. (2013). *Successful qualitative research: A practical guide for beginners*. SAGE.

Chawla, A., Chawla, R., & Jaggi, S. (2016). Microvasular and macrovascular complications in diabetes mellitus: Distinct or continuum?. *Indian journal of endocrinology and metabolism*, *20*(4), 546–551. https://doi.org/10.4103/2230-8210.183480

Craig, C. L., Marshall, A. L., Sjöström, M., Bauman, A. E., Booth, M. L., Ainsworth, B. E., Pratt, M., Ekelund, U., Yngve, A., Sallis, J. F., & Oja, P. (2003). International physical activity questionnaire: 12-country reliability and validity. *Medicine and science in sports and exercise*, *35*(8), 1381–1395. https://doi.org/10.1249/01.MSS.0000078924.61453.FB

Curtis, S., Gesler, W., Smith, G., & Washburn, S. (2000). Approaches to sampling and case selection in qualitative research: examples in the geography of health. *Social science & medicine*, *50*(7-8), 1001-1014.

CV, S. B., Balaji, S., & Seethalakshmi, A. (2012). Analysis of the degree of insulin resistance in post-menopausal women by using skin temperature measurements and fasting insulin and fasting glucose levels: a case control study. *Journal of clinical and diagnostic research: JCDR*, *6*(10), 1644.

Diabetes UK, (2021). Diabetes risk and waist measurement. https://www.diabetes.org.uk/preventing-type-2-diabetes/waist-measurement

Diabetes UK, (2022). Diabetes Distress. https://www.diabetes.org.uk/professionals/resources/shared-practice/psychological-care/emotional-health-professionals-guide/chapter-3-diabetes-distress

Diabetes UK, (2022). Diabetes self-management education. https://www.diabetes.org.uk/professionals/resources/resources-to-improve-your-clinical-practice/diabetes-self-management-education

Duboz, P., Chapuis-Lucciani, N., Boëtsch, G., & Gueye, L. (2012). Prevalence of diabetes and associated risk factors in a Senegalese urban (Dakar) population. *Diabetes & metabolism*, *38*(4), 332-336.

England, C., Thompson, J., Jago, R., Cooper, A., & Andrews, R. (2017). Development of a brief, reliable and valid diet assessment tool for impaired glucose tolerance and diabetes: The UK Diabetes and Diet Questionnaire. Public Health Nutrition, 20(2), 191-199. doi:10.1017/S1368980016002275

Fugard, A.J.B. & Potts, H. W. W. (2015). Supporting thinking on sample sizes for thematic analyses: A quantitative tool. *International Journal of Social Research Methodology, 18*(6), 669–684. https://doi.org/10.1080/13645579.2015.1005453

Global Adult Tobacco Survey Collaborative Group. Tobacco Questions for Surveys: A Subset of Key Questions from the Global Adult Tobacco Survey (GATS), 2nd Edition. Atlanta, GA: Centers for Disease Control and Prevention, 2011.

Greene, J. G. (1990). Factor analyses of climacteric symptoms: toward a consensual measure. Glasgow: *Department of Psychological Medicine, University of Glasgow.*

Greene J. G. (1998). Constructing a standard climacteric scale. *Maturitas,* 29(1), 25–31. https://doi.org/10.1016/s0378-5122(98)00025-5

Guest, G., Bunce, A., & Johnson, L. (2006). How Many Interviews Are Enough? An Experiment with Data Saturation and Variability. *Field Methods*, *18*(1), 59–82. https://doi.org/10.1177/1525822X05279903

Harlow, S. D., & Derby, C. A. (2015). Women's midlife health: Why the midlife matters. *Women's Midlife Health*, *1*, 5. https://doi.org/10.1186/s40695-015-0006-7

International Diabetes Federation (IDF), (2021). *What is diabetes?* https://www.idf.org/aboutdiabetes/what-is-diabetes/facts-figures.html

Information Commissioner Office (2022). The UK GPDR. https://ico.org.uk/for-organisations/dp-at-the-end-of-the-transition-period/data-protection-and-the-eu-in-detail/the-uk-gdpr/

Jehan, S., Masters-Isarilov, A., Salifu, I., Zizi, F., Jean-Louis, G., Pandi-Perumal, S. R., Gupta, R., Brzezinski, A., & McFarlane, S. I. (2015). Sleep disorders in postmenopausal women. *Journal of Sleep Disorders & Therapy*, *4*(5), 212.

Julious, S. A. (2005). Sample size of 12 per group rule of thumb for a pilot study. *Pharmaceutical Statistics: The Journal of Applied Statistics in the Pharmaceutical Industry*, *4*(4), 287-291.

Kalra, S., Jena, B. N., & Yeravdekar, R. (2018). Emotional and Psychological Needs of People with Diabetes.

Indian Journal of Endocrinology and Metabolism, *22*(5), 696–704. https://doi.org/10.4103/ijem.IJEM

Kautzky-Willer, A., Harreiter, J., & Pacini, G. (2016). Sex and Gender Differences in Risk, Pathophysiology and Complications of Type 2 Diabetes Mellitus. *Endocrine reviews,* 37(3), 278–316. https://doi.org/10.1210/er.2015-1137

Kiresuk, T. J., & Sherman, R. E. (1968). Goal attainment scaling: A general method for evaluating comprehensive community mental health programs. *Community mental health journal*, *4*, 443-453.

Lancaster, G. A., Dodd, S., & Williamson, P. R. (2004). Design and analysis of pilot studies: Recommendations for good practice. *Journal of Evaluation in Clinical Practice*, *10*(2), 307–312. https://doi.org/10.1111/j..2002.384.doc.x

Lee K. A. (1992). Self-reported sleep disturbances in employed women. *Sleep*, *15*(6), 493–498. https://doi.org/10.1093/sleep/15.6.493

Lizcano, F., & Guzmán, G. (2014). Estrogen Deficiency and the Origin of Obesity during Menopause. *BioMed research international*, *2014*, 757461. https://doi.org/10.1155/2014/757461.

Miles, M. B., & Huberman, A. M. (1994). *Qualitative data analysis: An expanded sourcebook*. sage.

Muka, T., Asllanaj, E., Avazverdi, N., Jaspers, L., Stringa, N., Milic, J., Ligthart, S., Ikram, M. A., Laven, J., Kavousi, M., Dehghan, A., & Franco, O. H. (2017). Age at natural menopause and risk of type 2 diabetes: A prospective cohort study. *Diabetologia*, *60*(10), 1951–1960. https://doi.org/10.1007/s00125-017-4346-8

National Institute for Health and Care Excellence. (2016). *Quality statement 2: Structured education programmes for adults with type 2 diabetes*. https://www.nice.org.uk/guidance/qs6/chapter/quality-statement-2-structured-education-programmes-for-adults-with-type-2-diabetes

Pearson JL, Hitchman SC, Brose LS, et al Recommended core items to assess e-cigarette use in population-based surveys Tobacco Control 2018;27:341-346.

Peters, S. A., Huxley, R. R., Sattar, N., & Woodward, M. (2015). Sex differences in the excess risk of cardiovascular diseases associated with Type 2 diabetes: Potential explanations and clinical implications. *Current Cardiovascular Risk Reports*, *9*(7), 36. https://doi.org/10.1007/s12170-015-0462-5

Pirolli, P., Mohan, S., Venkatakrishnan, A., Nelson, L., Silva, M., & Springer, A. (2017). Implementation intention and reminder effects on behavior change in a mobile health system: A predictive cognitive model. *Journal of Medical Internet Research*, *19*(11), e8217.

Polonsky, Fisher, L., Earles, J., Dudl, R. J., Lees, J., Mullan, J., & Jackson, R. A. (2005). Assessing psychosocial distress in diabetes: Development of the Diabetes Distress Scale. *Diabetes Care*, *28*(3), 626–631. https://doi.org/10.2337/diacare.28.3.626

Powers, M. A., Bardsley, J., Cypress, M., Duker, P., Funnell, M. M., Fischl, A. H., Maryniuk, M. D., Siminerio, L., & Vivian, E. (2016). Diabetes Self-management Education and Support in Type 2 Diabetes: A Joint Position Statement of the American Diabetes Association, the American Association of Diabetes Educators, and the Academy of Nutrition and Dietetics. *Clinical diabetes: a publication of the American Diabetes Association*, *34*(2), 70–80. https://doi.org/10.2337/diaclin.34.2.70

Prescott, J., Rathbone, A. L., & Brown, G. (2020). Online peer to peer support: Qualitative analysis of UK and US open mental health Facebook groups. *Digital Health*, *6*, 2055207620979209.

Public Health England. (2014). Adult obesity and type 2 diabetes. https://assets.publishing.service.gov.uk/government/uploads/system/uploads/attachment_data/file/338934/Adult_obesity_and_type_2_diabetes_.pdf

Richardson, C. D., Ray, G. J., DeWitt, M. A., Curie, G. L., & Corn, J. E. (2016). Enhancing homology-directed genome editing by catalytically active and inactive CRISPR-Cas9 using asymmetric donor DNA. *Nature Biotechnology*, *34*(3), 339–344. https://doi.org/10.1038/nbt.3481

Robinson, D. J., Luthra, M., & Vallis, M. (2013). Diabetes and Mental Health Canadian Diabetes Association Clinical Practice Guidelines Expert Committee. *Canadian Journal of Diabetes*, *37*, S87–S92. https://doi.org/10.1016/j.jcjd.2013.01.026

Shrivastava, S. R., Shrivastava, P. S., & Ramasamy, J. (2013). Role of self-care in management of diabetes mellitus. *Journal of diabetes and metabolic disorders*, 12(1), 14. https://doi.org/10.1186/2251-6581-12-14

Stewart, C., (2022). *Distribution of people registered with diabetes in England in 2020/21 by gender*. https://www.statista.com/statistics/387302/individuals-with-diabetes-by-gender-in-england-and-wales/

Sturt, J. A., Whitlock, S., Fox, C., Hearnshaw, H., Farmer, A. J., Wakelin, M., Eldridge, S., Griffiths, F., & Dale, J. (2008). Effects of the Diabetes Manual 1:1 structured education in primary care. *Diabetic Medicine*, *25*(6), 722–731. https://doi.org/10.1111/j.1464-5491.2008.02451.x

Sturt, J., Hearnshaw, H., & Wakelin, M. (2010). Validity and reliability of the DMSES UK: A measure of self-efficacy for type 2 diabetes self-management. *Primary Health Care Research & Development, 11*(4), 374-381. doi:10.1017/S1463423610000101

Teare, M.D., Dimairo, M., Shephard, N., Hayman, A., Whitehead, A., & Walters, S. (2015). Sample size requirements to estimate key design parameters from external pilot randomised controlled trials: A simulation study. *Trials, 2015*(15), 1–13.

Turner-Stokes, L. (2009). Goal attainment scaling (GAS) in rehabilitation: a practical guide. *Clinical rehabilitation*, *23*(4), 362-370.

Van Smoorenburg, A. N., Hertroijs, D., Dekkers, T., Elissen, A., & Melles, M. (2019). Patients' perspective on self-management: Type 2 diabetes in daily life. *BMC Health Services Research*, *19*(1), 605. https://doi.org/10.1186/s12913-019-4384-7

Ware, J. E., Jr, & Sherbourne, C. D. (1992). The MOS 36-item short-form health survey (SF-36). I. Conceptual framework and item selection. *Medical care*, *30*(6), 473–483.

Whicher, C. A., O'Neill, S., & Holt, R. I. G. (2020). Diabetes in the UK: 2019. *Diabetic Medicine, 37*(2), 242-247. https://doi.org/10.1111/dme.14225 [doi]

Whitehead, A. L., Julious, S. A., Cooper, C. L., & Campbell, M. J. (2016). Estimating the sample size for a pilot randomised trial to minimise the overall trial sample size for the external pilot and main trial for a continuous outcome variable. *Statistical Methods in Medical Research*, *25*(3), 1057–1073. https://doi.org/10.1177/0962280215588241

Wilcox G. (2005). Insulin and insulin resistance. *The Clinical biochemist. Reviews*, *26*(2), 19–39.

World Health Organisation (WHO) (2000). *Obesity: preventing and managing the global epidemic. Report of a WHO consultation*. In. Geneva

World Health Organization (2021). *Diabetes*. https://www.who.int/news-room/fact-sheets/detail/diabetes.

APPENDIX

Appendix 1: Demographic Questionnaire

DEMOGRAPHIC QUESTIONNAIRE

Study title: Women's Wellness Type 2 Diabetes Programme: A single arm trial of a Newly Designed Intervention for Midlife Women

Participant Name:	
Participant ID Code:	

**What is your age? (In years?)**	
**Country of residence?**	
**Country of Nationality?**	
**What sex were you assigned at birth, on your original birth certificate? (We would like to gain a fuller picture as to the healthcare needs of ALL midlife women living with type 2 diabetes to enable better and more informed care to be provided and to further equality.)**	
**What is your height, weight, waist&hip circumference and Boddy mass index (BMI)?**<br>**Please watch the video on the link for waist measurement** https://www.youtube.com/watch?v=e4cUSNq_OY8	
**How long have you had diabetes? (In years)**	
**Does anyone else in your family have diabetes?**<br>**Yes** ☐<br>**No** ☐	If **YES**, who?
**What is your relationship status? (Please tick the box).**	☐Single<br>☐Married<br>☐In a relationship

	☐Divorced<br>☐Widowed
**How do you describe your household's income?**<br><br>**(please tick the box)**	☐Poor<br>☐Lower-middle class<br>☐middle class<br>☐upper middle<br>☐wealthy
**How do you describe your ethnicity?**<br>**(Please tick the box).**	☐ White British ☐ Black Caribbean<br>☐ White others (Turkish)<br>☐ Asian ☐Black African<br>☐ Asian others ☐Black Other<br>☐ Chinese ☐ Mixed ethnic group<br>☐ Indian<br>☐Any other ethnic group; ____________
**What is the highest education qualification that you have completed? (Please tick the box)**	☐Primary school<br>☐Secondary school<br>☐College degree/Diploma<br>☐University/Degree<br>☐ Postgraduate degree<br>☐Others (write in) ___________
**Are you currently working? (Please tick the box)**	☐Employed full-time<br>☐Employed part-time<br>☐Home duties

	☐Unemployed<br>☐Student<br>☐Unable to work<br>☐Others (write in) ___________
**How do you manage your diabetes? (Please tick the box(s) or write in)**	☐No treatment<br>☐Diet and/or exercise<br>☐Insulin and/or other injections for diabetes<br>☐Tablets<br>☐ A Different diabetes management method (please write what treatment is); ___________
**What was your last long-term blood glucose test result and when (also called HbA1c or A1C)? (If known)**<br>**You can find this information on your NHS App or from your GP.**	
**Has your diabetes caused any of following problems? (Tick as many issues as apply)**	**Diabetes issues**<br>☐ Heart Disease<br>☐ Leg Ulcers<br>☐ Numbness or pain in your hand or legs<br>☐ Problems with eyesight<br>☐ Kidney problems<br>☐ Menstrual problems<br>☐ Polycystic ovary syndrome<br>☐ Menopause problems<br>☐ Anxiety

	☐ Depression<br><br>☐ Please specify other health issue(s) if you have; ___________
**Have you ever attended a diabetes education course? If yes, what type of course, please?**	☐ Please identify if you have attended _______

Thank you for completing this questionnaire and for considering taking part in this research.

Email: deniz.bozkurt@kcl.ac.uk

Appendix 2: Interview Topic Guide

Interviews will be conducted via Microsoft team 30 to 45 minutes or 45 to 60 minutes.

Discussion on the peer support group and handbook

1. Did you read the guidance handbook? If no, can you tell me why? It's okay if you don't like to. If yes, what did you think about its content?
2. Please could you tell me what was it like to be in the peer support group?
3. Can you tell me about what went well for you in the group? Can you give me some examples? How can we do more of that?
4. How about the things you found challenging? How did you overcome them? How can we make it better?

Discussion on the Website and its content

5. Which elements did you find easy to go through and why?
6. Which parts you think could be better? How?
7. Can you tell me how the program website and its content might help to improve your type 2 diabetes?
8. Would you like to continue to access the program? If Yes or No, why? What would make you choose differently?
9. During programme duration, did you get any healthcare or support from NHS for your type 2 diabetes? Can you give me some examples? (Without naming names)

Discussion on Personal Goal Evaluation Process

10. How do you feel about the goal setting form? Can you give me examples of which parts you liked?
11. What about the parts that you think could be better?
12. Can you tell me how did choose your goals? What made you prioritise them in that order? How did that step make you feel?

And so on…

13. if someone else has type 2 diabetes and they ask for your opinion on the program, what would you say?
14. What would you like to add to our discussion?

Topics

Peer support group

The program website and content

Personalised goal evaluation form

Other healthcare support during program

Prompts: Log into or accessing to website, Navigation of website, WWDP terms and conditions, Log Book, Content of eBook (Resources), Podcast, Technical issues, Feedback/Support tool, FAQs, Automatic emails weekly by website, Certificate for completion of WWDP.

General prompts

Can you tell me a bit more about that?

Why do you think?

Can you please give examples?

What are your thoughts on that?

What do you mean by …?

How so?

In what way will it work well for you?

Appendix 3: Participant information Sheet

WE INVITE YOU TO TAKE PART IN A RESEARCH STUDY

- The Women's Wellness with Type 2 Diabetes Programme (WWDP) is a 12-week structured lifestyle program. The WWDP offers an e-book, a peer support group and two peer supporters to help women with type 2 diabetes to achieve a healthy lifestyle, and better confidence with managing their diabetes.
- Before you decide whether to take part, it is important for you to understand why the research is being done and what it will involve.
- Please take time to read the following information carefully. Discuss it with friends and relatives if you wish.
- You are free to decide not to take part. This will not affect the care you get from your own doctors.
- Please ask us if there is anything that is not clear or if you would like more information.

WHAT IS THE PURPOSE OF THE STUDY?

Feeling and living well is key to having a healthy life with type 2 diabetes. Diabetes can lead to many health problems in the eyes, kidneys, nerves, and circulation if it is not well managed. Diabetes can result in people being emotionally overwhelmed by the demands of managing it. The Women's Wellness with Diabetes Programme aims to help midlife women to feel well in their life and approach their diabetes positively so that these complications and negative feelings are avoided. The Women's Wellness with Diabetes Programme has merged two successful programmes called The Diabetes Manual and the Women's Wellness programme for midlife women. This research will evaluate what midlife women with type 2 diabetes think of this newly designed programme and if it improves their physical and emotional wellbeing as well as explore your experiences with the content and delivery of the new online intervention. The study will be carried out in the UK. We will see whether women join the programme, stick with it and are happy to complete the study questionnaires and how it impacts on their health. This small study will help us plan for a bigger study to see whether the WWDP should be offered by the NHS.

WHY HAVE I BEEN INVITED?

All individuals who identify as a woman are eligible to take part in this study because they have type 2 diabetes and are a woman aged between 45 to 65 years. We want to invite up to 40 women who are already feeling emotionally stressed about their diabetes.

DO I HAVE TO TAKE PART?

It is up to you whether to join the study. If you agree to take part, we will ask you to sign a consent form. A research assistant will contact you after 24 hours receiving your email about that includes you would like to participate to study or filling the survey on the KCL website. They will contact you by phone to address any questions and confirm eligibility, you will then complete a consent form online.

WHAT IF I CHANGE MY MIND ABOUT TAKING PART?

For study 1 (questionnaires), you are free to withdraw without giving a reason until end of day 10 following the survey completion. *For study 2* (interviews), if the interviews have been conducted, you will have ten days post interview to withdraw your data. After these timeframes, we will begin data analysis and it will not be possible to remove your data from the study. If you wish to withdraw from the study and request that your data, be withdrawn will be able to reach the principal investigator through the email provided at the bottom of the information sheet.

WHAT WILL HAPPEN TO ME IF I TAKE PART?

Firstly, you will fill out the survey called screening questionnaire that asks your age, your type of diabetes, residency, your stress level of diabetes etc...on the KCL website. After your eligibility confirmed, the researcher will contact you via email or

by phone in 24 hours. Then, you will receive another online survey link to be completed in 7 days for consent form with baseline questionnaire that includes demographic questionnaire such as health and life information (for example age, income, ethnicity and your time since diabetes diagnosis) and standard questionnaires including diet, diabetes distress, physical activity, menopause, sleep quality, quality of life as well as self-efficacy prior to intervention. This will also include measuring your weight and waist circumference by yourself. If you need more information about how to measure waist circumference, you will find a video link on the questionnaire align with this question. Once this has been completed, you will receive a Women's Wellness with Diabetes Programme website log in details for your electronic devices or smartphones.

Women's Wellness Type 2 Diabetes Programme is a 12-week program that will help you a) understand more about your diabetes, b) set in place positive lifestyle changes and habits to make a difference to your overall health and wellbeing. There are four steps to this program with each step taking three weeks to complete. In step 1, we talk about type 2 diabetes, get you started on the program and provide you with practical healthy lifestyle information to strengthen your confidence and motivation. You will complete a personal goal/s form which is a self-guided tool to help you set up your goals regarding your diabetes health habit interests. Step 2 is about consolidating the changes you have made in the first few weeks through practising and self-monitoring your new habits. In step 3, you will learn about important health issues for midlife women, and you will be encouraged to maintain the healthy lifestyle habits you have started. Finally in step 4, we want you to retain the confidence and motivation you have to develop your own strategies to maintain your healthy lifestyle behaviours for the rest of your life. The intervention includes an online website (to log in from any smart device using the User ID and password), eBook (weekly reading in order of different content related to their type 2 diabetes health journey for 12 weeks), Factsheets (include different topics on key information on type 2 diabetes, Logbook (to save and make notes of your daily activities intervention duration). You will be expected to enter the website weekly to read the eBook that includes information and record the daily activities on your e-Logbook which only you have access to.

One of the topics on eBook is exercise and you will read about exercise recommendations in the physical activity section. You make commence exercise activity that is too strenuous at first and feel out of breath or have sore muscles afterwards. If you have any concerns about commencing or continuing with physical activity during the programme, please talk to your diabetes nurse or GP. You can listen to podcasts related to your own specific interests and participate in an online peer support group through a private Facebook group. The personal goal form is a self-guided tool that will help you to set up your goals regarding your interest on type 2 diabetes during intervention. You will be expected to choose your 1 or 2 goals independently; however, the online content on the WWDP website, of weeks, 1, 2 and 3 of eBook will support this process.

WWDP WEEK 1-PREPARATION you will read information to understand your diabetes, self-care activities, and goals. Following this, WEEK 2-3 CHANGING LIFESTYLE will provide information about healthy eating with type 2 diabetes, understanding more about glucose, alcohol and smoking, healthy shopping, and meal planning, changing eating behaviours, stretching and flexibility, strength training exercise, diabetes distress and strategies for managing stress, better sleep, and goal setting recommendations. On the first day of the WEEK 3, you will receive the Personal Goal Form via email, and you will be expected to independently set up 1 or 2 Goal at the end of the WEEK 3; to have discussions about how everyone is approaching this goal setting and form completion. Overall, you will be ready to set up your goals using the personal goal form instructions at the end of week 3.

The peer support group is an online private Facebook group (12) comprising two 2 moderators who will facilitate Monday to Friday between 9:00 am to 5:00 pm. The moderators are volunteer midlife women living with type 2 diabetes who have been purposely fully recruited through Diabetes UK and have undertaken five hours of training. The moderators will be supervised by the PI and supervisors Prof Sturt, Dr Duaso and Dr Nadal. The peer support group will help you to engage with, and learn from, other participants during 12-weeks.In this space, you can share and learn from others' experiences, post questions, and discuss topics related to weekly content provided on our website. While the group is moderated to ensure a supportive environment, please be aware that real-time interaction is not guaranteed. To enrich your experience, you are encouraged to participate in activities such as quizzes, message boards, and direct questions at your convenience. You are expected to post or comment at least three times a week to foster a vibrant community dialogue.

To join the group, you will receive an email invitation by the research team. Upon requesting access to the Facebook group, you'll need to enter a unique ID number provided to you. Before participating, we will ask you to review the peer

support guidelines on the resources page of our website. You will be asked whether you would like the FB group to remain open, unmoderated, following the study. Please see below chart for a summary of intervention process 12 weeks.

At the end of the study, there will be an optional feedback interview with the researcher. Interview participants will be purposively selected based on your engagement with the program. Minimum requirements to engage with the programme for 12 weeks; Log in to website ≥3 times and attendance of peer group ≥3 times. This feedback interview will take place by video call and be recorded via Microsoft Teams program. The research team will help you to access these. This record will be transcribed as given unique ID number and then deleted.

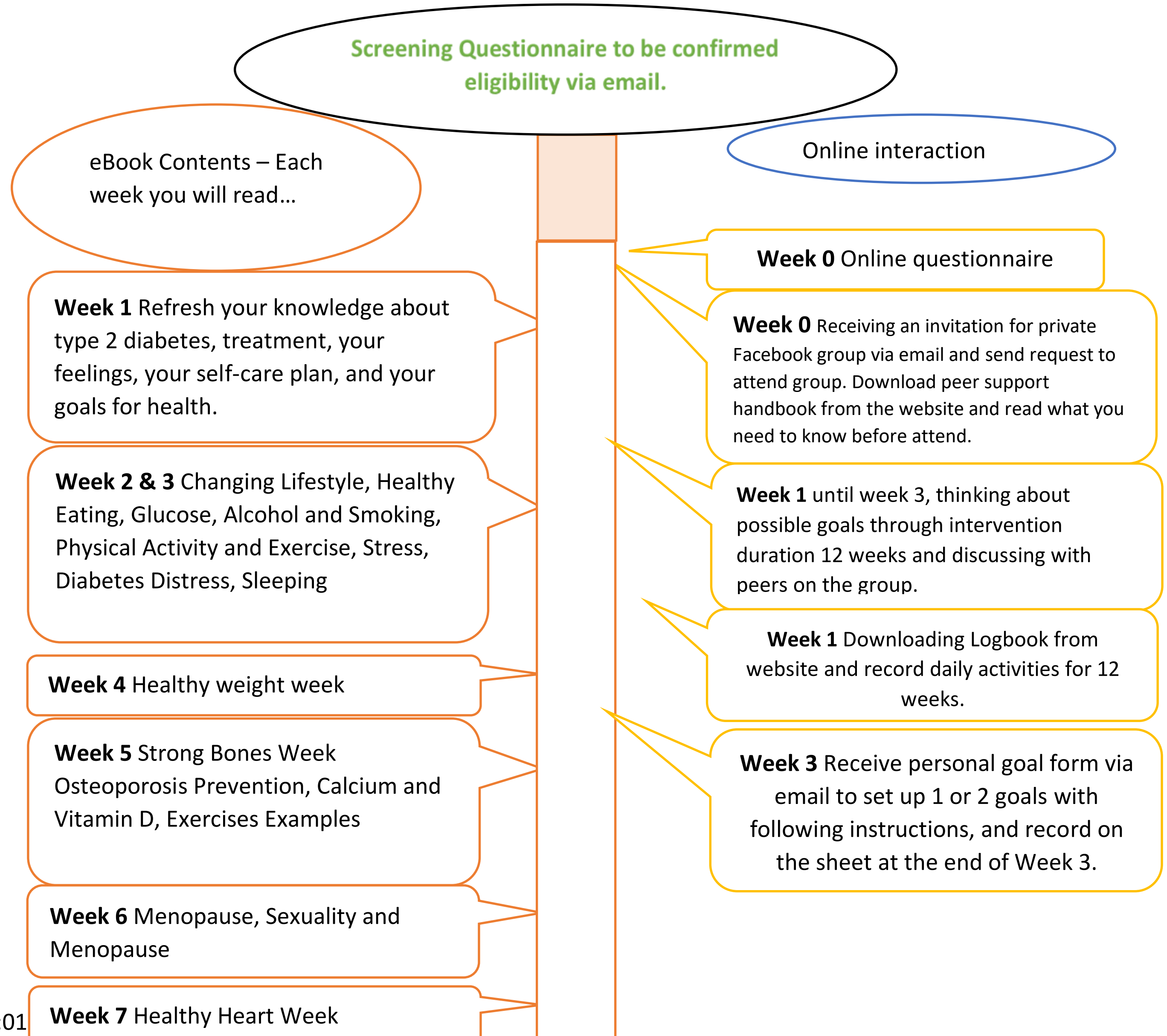

Week 8 Diabetes Complications Prevention Week, Your regular tests and check-ups, Preventing complications of diabetes, Short and long term complications

Week 9 Cancer Prevention Week

Week 10 Reviewing what you have learned

Week 11 Motivation and change, Overcoming obstacles to change

Week 12 Looking forward – goal setting

Week 12 Online questionnaire including reviewing goal setting process and evaluation.

Week 12 Receiving consent form via email for feedback interview via Microsoft Teams.

Week 16 Interviews will be conducted as approximately 60 minutes via Teams.

Can I take Breaks during the 12 week program? Is it possible?

Sometimes life gets in the way of our newly developing habits and healthy lifestyle behaviours. For example, this might be because we take a holiday, we have a celebration, we get busy at work, or someone gets ill. The Women's Wellness with Diabetes Programme is realistic about these life events, and we will ask you before you start whether you are expecting any of these events. If you are we will suggest you commence the programme once the event is over. For unexpected events, the programme can accommodate a break of up to two weeks.

As it is a research study, we hope that everyone will be able to complete the WWDP by the time the study ends. However, if you have not been able to do so because of personal events like those above, you will have unlimited access to the workbook and access to the website until week 14 even though we may not be collecting information from you.
During any periods when you take a break from the 12-week programme, you will retain access to the peer support FB group and will be encouraged to continue your engagement with it.

Can I attend routine appointments with my GP and other Health Care Professionals?

Yes, you should continue to attend any routine appointments with your usual healthcare providers and that this program is not intended to replace any treatment they are currently receiving for your type 2 diabetes.

I do not have a Microsoft Teams account and Facebook account.

The researcher will help you to set up an account and show you how to use it.

Do I get paid?

This is voluntary. There is no payment available.

WHAT ARE THE POSSIBLE DISADVANTAGES AND RISKS OF TAKING PART?

Physical Risk for Intervention: The use of the intervention is associated with potential risks. It is important to be aware of any possibilities for injury during exercise, especially if you do not exercise regularly. To minimise the risk of injury from exercise, intervention programs depend on the usage as much as you could as individual. The intervention advises you gradually increase your exercise levels over time, you do not overexert themselves at the beginning. To prevent stiffness and promote flexibility, you are also encouraged to stretch regularly. Due to your involvement in reading the eBook every day, there may be a risk of tiredness in your eyes, and stiffness in your shoulders. During the program, you might like to incorporate some exercise into your daily routine, which could potentially reduce this risk.

Psychological risks for intervention: The topics that you could find uncomfortable the section on cancer and type 2 diabetes related complications. You may choose not to speak about topics or questions that make you uncomfortable.

Psychological risks for peer support group: Online moderated peer support groups aim to provide a supportive environment, but there are still potential risks. Online communication can be prone to misinterpretation and misunderstandings. Failure to address harmful or disruptive behaviour can lead to a decline in the quality of the group and your well-being. To mitigate this, we will ensure moderators have adequate expertise, establish clear guidelines, and prioritise participant well-being.

Other risks: Study 2 involves midlife women with T2DM, and interview topics cover general, health and diabetes questions. There is no evidence to suggest that participating will be upsetting or distressing. You may choose not to speak about topics or questions that make you uncomfortable or that you can stop participating at any time without giving a reason.

If you feel uncomfortable with a question or any topic in the eBook, you do not have to answer every question or read the topic. If you have any concerns about research, please email to the researcher. Support resources:

If you are experiencing distress and would like to receive support from a health professional, please contact following resources:

1. Share your concerns making you feel distressed on the peer support online group. The purpose of the group is to facilitate peer support when things are feeling tough and when you experience success.
2. Your usual diabetes provider: They provide a medical diabetes examination, healthcare, diabetes symptom management and when necessary, your diabetes provider can refer you to Mental Health Service for further examination, support, and care.
3. Diabetes UK: Diabetes UK has trained counsellors who provide a free and confidential talk about uneasy emotions. You can get in touch with their highly trained advisors for support, advice or just a chat. You can tell their helpline on 0345 123 2399, Monday to Friday, 9 am to 6pm.
4. Samaritans: It's a 24-hour service that provide confidential telephone support for adults who have difficulties with their emotions. Helpline: 116 123 (UK).

Peer supporters are facilitator and moderator of the online peer support group. Your facilitator is a Peer support, when one or more people like you offer you information, encouragement, and reassurance, is well known to be effective in helping people cope with difficult or challenging situations. Peer support has been used in diabetes communities for decades. Peer support can be even stronger when peers are more like each other in gender, age, and health circumstances for example. The private Facebook peer support group aims to offer this type of support from other women very similar to yourself. Thus, you will be able to see how other women living with type 2 diabetes cope with the management of it in their life and this will guide each other during the peer group. Peer supporters are facilitators and moderators of the online peer support group. Your facilitator is a non-professional who is between 45-65 age range and living with type 2 diabetes and will provide you with support as motivation during your time on the programme by online messaging. Your facilitator will be able to see your Facebook profile. In order to ensure that their support is relevant and specific to you, and to ensure your wellbeing, your facilitator will be able to see your messages and posts so your conversation on the peer group and i.e. your goals and process.

However, if you think your concern is likely to be best addressed by a health professional, then they are the best people to contact. If you share your distress, questions, or concerns on the peer support group, peers or the moderator may suggest that you contact your GP or diabetes nurse.

WHAT ARE THE POSSIBLE BENEFITS OF TAKING PART?

If you complete the 12-week program, the earlier Women's Wellness programmes found women experienced: more knowledge about their general health improved health related quality of life; decreased BMI; a better understanding of your diet; a habit of regular exercise. We expect that this programme with peer support group will also leave you with more knowledge and confidence to manage your diabetes.

WILL MY TAKING PART BE KEPT CONFIDENTIAL?

With your consent, your participation will be confidential.

All identifiable participant data will be de-identified and stored in line with the Data Protection Act 1998 in the UK and non-identifiable data for Australia and provider and developer DAWN for WWDP. (DAWN is a health research community that brings together all the research, training, and wellness programs being developed by research teams across the world. It is hosted and led by the Women's Wellness Research Collaborative in Australia). The research team manage the WWDP website; however, any technical issues on the website will be resolved by the Dawn IT team. Your confidential data will be stored on a secure server at King's College London research team can access these data. A separate electronic file will hold the identifiable details and access to this will be restricted to 4 members of the King's College London university research teams.

WHAT WILL HAPPEN TO MY DATA IF I WITHDRAW FROM THE STUDY?

With your consent, we will keep and use the data provided. The data will be anonymised. After the specific period, it will no longer be possible to withdraw from the study because the collected data will be anonymised and will be included in the analysis.

HOW IS THE PROJECT BEING FUNDED?

This project is funded by a doctoral fellowship for the main researcher by the Ministry of Education, Turkey.

WHAT WILL HAPPEN TO THE RESULTS OF THE STUDY?

We aim to publish the results of this study in peer reviewed scientific journals. It will not be possible to identify any individual participant from the published data. If this study goes well, then we will seek funding to conduct a larger study in the future.

WHO CAN I CONTACT FOR MORE INFORMATION?

Researcher: Deniz Bozkurt email: deniz.bozkurt@kcl.ac.uk

Address: King's College London, Florence Nightingale Faculty of Nursing, Midwifery & Palliative Care James Clerk Maxwell Building Room 1.32, 57 Waterloo Road LONDON SE1 8WA

WHAT IF I HAVE FURTHER QUESTIONS, OR IF SOMETHING GOES WRONG?

Supervisor: Dr Maria Duaso, maria.duaso@kcl.ac.uk
Address: Florence Nightingale Faculty of Nursing, Midwifery & Palliative Care King's College London Room 3.19, JCMB LONDON SE1 8WA

Supervisor: Prof Jackie Sturt, Jackie.sturt@kcl.ac.uk
Address: Florence Nightingale Faculty of Nursing, Midwifery & Palliative Care King's College London Room 4.30, JCMB LONDON SE1 8WA

Supervisor: Iliatha Papachristou Nadal iliatha.papachristounadal@kcl.ac.uk
Address: Florence Nightingale Faculty of Nursing, Midwifery & Palliative Care King's College London

Appendix 4: WWDP terms and conditions

Welcome to **WWDP: Women's Wellness Type 2 Diabetes Programme**

Before we get started we would like to go through what you can expect from the WWDP.

The WWDP Agreement

Throughout WWDP, you will be supported by the principal investigator. The principal investigator will contact you after you are interested in participating in the WWDP. Facilitator who is peer supporter will see all messages and support in the online peer group.

Below is an Agreement which outlines what you and your facilitator will do during the programme.

<u>Our Agreement</u>

Me

- I understand that WWDP is not an emergency programme. If I am feeling very distressed or need immediate help I will contact friends or family who can help, or my GP.

- I understand that my facilitator will regularly see my messages during peer group, but they may not be able to respond to messages immediately.

- I understand that the principal investigator and facilitator is able to see my messages through the programme. This will help them provide the most relevant support for me.

- I understand that I will have access to facilitator support for 12 weeks from the time I register and have access to the WWDP. Four months after I have registered, my access will expire.

- I understand that I may receive reminder emails or messages from the programme.

- If technical issues arise, I understand that the IT support team may see my email address. All other personal information is encrypted and cannot be viewed.

Principal Investigator

- Will keep my personal contact information private.

- Will ask me some questions before enrolling me to the WWDP in email or an initial 30-minute telephone call

- Will review my progress regularly.

Peer Supporter (Facilitator=Moderator)

-Will see my conversation through the online messaging platform thereafter.

Data Security and Privacy

OVERVIEW

In this section we outline the data processes that happen as part of WWDP and explain what data will be collected and what happens to that data.

Data security and confidentiality are a priority for WWDP.

As well as the data security processes we outline below, WWDP will log you out automatically after 2 hours of inactivity.

We also recommend:

- logging out whenever you have finished working on WWDP
- keeping your WWDP username and password in a secure place
- not sharing any unnecessary personal information or contact details with your facilitator via your notes or online messages (this is explained in more detail below)

Device security

Your security and privacy on WWDP depend on the device you use to access the website. To ensure maximum security, we recommend you keep your software up-to-date, choose strong passwords which are not easy to guess, use security protection software and avoid unsecured public internet connections.

Who developed WWDP?

WWDP was developed by a team of researchers at King's College London University and Griffith University. You will have a new designed WWDP by principal investigator at King's College London and works in partnership with:

1. DAWN – a software development company who programmed DAWN. DAWN hosts and maintains the website. DAWN meets NHS Digital standards for privacy, confidentiality, and security.

PERSONAL DATA

What personal data/information will WWDP ask me for?

WWDP is a website. So that you can use WWDP, we need you to register with an email address. WWDP is part of a research study. This means that we may also ask for the following information:

1. Your name
2. Your date of birth
3. Your address

4. Your telephone number
5. Your gender

Why does WWDP need to collect this personal information?

Because peer support group of WWDP is part of a research study, the research team needs accurate and up-to-date information. Collecting this information means that your principal investigator can monitor your progress.

Where are my data kept?

The WWDP website is hosted by DAWN. This means that the data collected by WWDP is held on a database managed by DAWN. This database is located in a securely protected and approved provider cloud solution. When you register for WWDP you are connected to a name and an email as you need the name to set up a portal login. Additionally, because your data is confidential, the data that WWDP collects is held in an encrypted state.

Who can access my personal information?

We have to follow strict privacy, confidentiality, and online security procedures.

Your personal information will only ever be accessed by the research team. WWDP will never share your information with other parties without your written consent. For example, if access is needed to your WWDP account due to a technical error we will ask for your written consent for a member of the technical team to do this.

Does WWDP collect other information about me?

Every account has a username, email, first name, and last name to log in to the portal. There will be not data sharing between KCL and DAWN.

Does WWDP share my information with anyone else?

If you have a technical problem or question and you submit a question/concern via the Contact us page, then King's College London will receive the following information

1. Your email address
2. Information you type in your message

In order to solve a technical problem, support from the DAWN software team who programmed WWDP may be needed. We will never share your information with DAWN or ask them to look into the problem without gaining your informed consent first.

WWDP will never share your personal information with anyone without your consent.

WWDP - Contact Us

Links&Podcasts

Some sessions or podcasts may contain links to other websites which are owned, operated or maintained by third parties. If you click on a third-party link, you will be directed to that website in a new tab. We provide these links as helpful sources of further information, not as an endorsement, authorisation or representation of our affiliation with that third party, nor as an endorsement of their privacy or information security policies or practices. We do not have control over third party websites and we do not have control over their privacy policies and terms of use.

Who can see what I write in WWDP?

When you join WWDP, you will see peer supporters as facilitator. Your facilitator is a non- professional who is between 45-65 age range and living with type 2 diabetes and will provide you with support as motivation during your time on the programme by online messaging. Your facilitator will be able to see your name on the support group.

In order to ensure that their support is relevant and specific to you, and to ensure your wellbeing, your facilitator will be able to see your messages so your conversation on the peer group and i.e. your goals and process.

Principal investigator will be able to review your online messages with your facilitator and group. The content of these messages will be kept confidential at all times.

The WWDP Team at King's College London

Chief Investigator for WWDP: RN, MSc Deniz Bozkurt

Academic supervisors: Dr Maria Duaso

Professor Jackie Sturt

The legal bits

Information collected by WWDP will be in line with the General Data Protection Regulation (2018). Our lawful basis for collecting this information includes:

1. Function of a public task
2. Vital interest
3. Legitimate interest
4. Consent

Your rights

Your personal data will be processed in accordance with your rights under data protection legislation.

Your rights are:

a. right to be informed
b. right to gain access to your data
c. right of rectification (e.g. change inaccurate information)
d. right to erasure (e.g. to delete records held about you on the WWDP platform)
e. right to restriction (e.g. to stop processing information about you)
f. right to portability (e.g. to move or transfer your data)
g. right to object (e.g. to change your mind)
h. right not to be subject to automatic profiling or decision making (e.g. to know if a decision was made by a computer rather than a person)

Cookies

The WWDP website does not use marketing cookies. This means that your browsing information is not used for advertising or commercial purposes. The WWDP website does use one type of cookie – this is an essential cookie which is used to make sure you get a consistent user experience. It is required for the website to maintain where you are up to in the programme. It also means it can show you personalised information when you are signed in.

SUMMARY

Your personal information will be managed and shared in line with the General Data Protection Regulations (2018) and common law duty of confidentiality.

1. WWDP will ask for personal information. This information will be stored in line with GPDR data privacy and security standards.
2. WWDP is developed by King's College London and owned by Professor Debra Anderson and Professor Jackie Sturt.
3. WWDP will not share any of your data WWDP.
4. Filling in the WWDP contact us form, means your email address and typed message will be seen by the King's College London team.
5. If you experience a technical problem, the WWDP team at King's College London will respond to your concern and gain your consent for the web-developers of WWDP to access your information.

If you have any concerns or further questions, please contact the WWDP team using the form which you can find in the Contact us page.

You can find more tips for staying safe online at www.cyberaware.gov.uk.

The following video also provides a useful overview of patient data - vimeo.com/264239790

WWDP - Contact Us

Please tick the box to show that you have read the Agreement and understand the terms of using WWDP.

If you do not agree to this, please contact the WWDP team using the form which you can find in the Contact Us page.

Appendix 5: Personal Goal Form with GAS

Personal Goals Form

Prioritising a behavioral goal relating to your diabetes and overall health.

Welcome to your goal setting session. Below you will find four questions which will help you to set up your goals as you begin the WW2DPduring intervention. Today we ask you to choose one goal from the list below and be expected to choose **1** or **2 goals** (which is most important to you) during **intervention session.** You can have up to five days to consider this and choose your first goal priority. Once you have chosen your goal, please complete the remaining questions 2-5. You will be asked to complete this Personal Goal Form again at the end of the WW2DP in week 12. Please do not forget to check your email junk box towards the end of the WW2DP if you have not received it. Each form will have a unique identification number and not include any identifiable participant information.

1) Read the list of 8 diabetes self-care activities below. Think about which of these activities you would like to do, even if you are not doing them now. You can take up to five days to think about this. Next place a number next to activities that are most important to you for your health. Number 1 is most important, and number 8 is least important.

	Weight and body mass index

	Eating a healthy diet
	Taking regular physical activity
	Reducing unhealthy habits
	Reducing your stress
	Managing menopause and its symptoms
	Taking steps to help you sleep well
	Diabetes specific healthy behaviours e.g<br>• Monitoring your own blood glucose (sugar) levels regularly (even if you cannot do this currently)<br>• Taking your diabetes medication<br>• Attending diabetes appointments for your eyes and foot checks and blood tests

2) Rate the level of confidence you have in making changes in any of these self-care activities.

Activities of diabetes self-management	Lowest	Somewhat	Moderate	Good	Most
Weight and body mass index	1	2	3	4	5
Eating a healthy diet	1	2	3	4	5
Taking regular physical activity	1	2	3	4	5
Reducing unhealthy habits	1	2	3	4	5
Reducing your stress	1	2	3	4	5
Managing menopause and its symptoms	1	2	3	4	5
Taking steps to help you sleep well	1	2	3	4	5
Diabetes specific healthy behaviours	1	2	3	4	5

3) Think about how much motivation you have for making changes in any of these areas. Please circle the number which matches your motivation levels for each of the diabetes self-care activities.

Activities of diabetes self-management	Lowest	Somewhat	Moderate	Strongly	Most
Weight and body mass index	1	2	3	4	5
Eating a healthy diet	1	2	3	4	5
Taking regular physical activity	1	2	3	4	5
Reducing unhealthy habits	1	2	3	4	5
Reducing your stress	1	2	3	4	5
Managing menopause and its symptoms	1	2	3	4	5
Taking steps to help you sleep well	1	2	3	4	5
Diabetes specific healthy behaviours	1	2	3	4	5

4. Family and friends can often want us to change things about our health. Think about what your family and friends have talked to you about and where they are motivated to help you make changes.

Activities of diabetes self-management	Lowest	Somewhat	Moderate	Strongly	Most
Weight and body mass index	1	2	3	4	5
Eating a healthy diet	1	2	3	4	5

Taking regular physical activity	1	2	3	4	5
Reducing unhealthy habits	1	2	3	4	5
Reducing your stress	1	2	3	4	5
Managing menopause and its symptoms	1	2	3	4	5
Taking steps to help you sleep well	1	2	3	4	5
Diabetes specific healthy behaviours	1	2	3	4	5

Finalising your personal goal priority is a balance between your motivation and level of confidence you believe have for making changes in these activities. The support of those around you for achieving particular goals can also be important. The **higher your level of motivation** and the more confidence you believe you have, the more likely it is that you will be successful **in achieving your goal.** You may also wish to consider the views of those around you on your health priorities.

Now you have had a chance to think about all these important aspects of setting goals for yourself, take another look at your priorities in question 1 and repeat the scoring in the table below (question 5)

5) Which of the following diabetes self-care activities do you feel is **the most important to you.** List from highest priority = 1 and the lowest =5.

	Weight and body mass index
	Eating a healthy diet
	Taking regular physical activity
	Reducing unhealthy habits
	Reducing your stress
	Managing menopause and its symptoms
	Taking steps to help you sleep well
	Diabetes specific healthy behaviours;<br>• Monitoring your own blood glucose (sugar) levels regularly (even if you cannot do this currently)<br>• Taking your diabetes medication<br>• Attending diabetes appointments for your eyes and foot checks and blood tests

Please write down the most important goal priority for you here………………….

Personal Goals Form:

*Developing your personal goal is a balance between your motivation and the level of confidence you feel you have over the activities. The **higher your motivation** and the higher your level of confidence the more likely it is that you will be successful **in achieving your goal**.*

Instructions: Now that you have chosen your first goal we would like you to think about it in more detail. Read the information below as an example of the type of detail that is important. The more detail you think about, the more likely you will be able to achieve your own goal.

Table 1: An example Personal Goals Form with instructions

Goal name and timeline	Score	
Please write here your first goal name and when you want to achieve it by (your timeline)		
Oral (Mouth) health: I will brush my teeth and gums twice a day		

Table 1: An example PGF evaluation form within instructions

**GOAL ATTAINTMENT LEVEL**	**SCORE**	**GOAL NAME&TIMELINE**
		Please write here your first goal name and when you want to achieve it by (your timeline)
		Please explain **what you would like to** and **how would like to achieve** through WWDP. For example, I would like to reduce unhealthy habits. I will choose a topic related to my goal from eBook (Please look the menu on the http://www.wwt2dp.co.uk/website) and reading it daily and discussing my struggles with peer group.<br><br>Please see below example of another table 2 setting up sentences for goals.
Much better than expected	+2	Then you will write here or one of the below scores that **how much you achieved than expected** related to your goal process at the end of the session
Somewhat better than expected	+1	

Expected level of attainment	0	
Somewhat less than expected	-1	
much less than expected	-2	

Table 2 Sample goal statements and GAS

Goal attainment level	Score	Goal areas			
		Goal 1	Goal 2	Goal 3	Goal 4
		Oral health	Stress reduction	Physical activity	Healthy diet
		I will brush my teeth and gums three times a day for myself.	I will find some time and energy for recreational activities once per week.	I will establish a regular, routine fitness program three times per week.	I will increase intake of fruits and vegetables by one serving per day.
Much better than expected	+2	I brushed my teeth and gums more than four times a day for myself.	I reserved some time and energy for recreational activities several times each week.	I exercised more than four to five times per week.	I increased intake of fruits and vegetables to more than two servings per day.
Somewhat better than expected	+1	I brushed my teeth and gums four times a day for myself.	I reserved some time and energy for recreational activities more than once per week.	I exercised four to five times per week.	I increased intake of fruits and vegetables by two servings per day.
The expected level of attainment	0	I brushed my teeth and gums three times a day for myself.	I reserved some time and energy for recreational activities once per week.	I established a regular, routine fitness program three times per week.	I increased intake of fruits and vegetables by one serving per day.
Somewhat less than expected	−1	Brushing less than three times a day for myself.	No change in time and energy resources related to recreation.	Exercise less than three times per week.	No change in fruit and vegetable intake.
Much less than expected	−2	Brushing less than two times a day for myself.	Less time and energy resources reserved for recreation each week.	Very little or no exercise.	Decreased fruit and vegetable intake.

Abbreviation: GAS, Goal Attainment Scaling.

Table 3: PGF EVALUATION FORM

GOAL ATTAINTMENT LEVEL	SCORE	GOAL NAME&TIMELINE
Much better than expected	+2	
Somewhat better than expected	+1	
Expected level of attainment	0	
Somewhat less than expected	-1	
much less than expected	-2	

the table below above and how you would like to achieve align with www.wwt2dp.co.uk (Women's Wellness Type 2 Diabetes Programme website (WWDP)) and what you expect to achieve in that time (setting up a timeframe). For example, **Goal:** Managing my stress **Timeline:** 12 weeks. Timeline will depend on your flexibility; however, intervention duration is twelve (12) weeks, and you will be expected to choose 1 or 2 goals during intervention session. At the end of session, then you will review your goal process whether it's much better than expected or much less and choose one of the scores and explain how you managed it align with WWDP and peer group and will return this form to researcher via email. Please see below an example filled form for **Table 1 Personal Goal evaluation form** and **table 2** before starting to fill form in **Table 3**. Form areas are flexible and there is no limitation of words to write on it. If you have any problems to fill the form or access it, please send an email to researcher deniz.bozkurt@kcl.ac.uk

NOTE: Please return this form at the end of session once you completed to the researcher via email deniz.bozkurt@kcl.co.uk Thank you for completing this form and returning it.

Appendix 6: A general view of intervention website and new factsheets

A. Healthy eating and cooking with T2DM and Managing celebrations with T2DM

APPENDIX

Appendix 7: *A summary of Intervention*

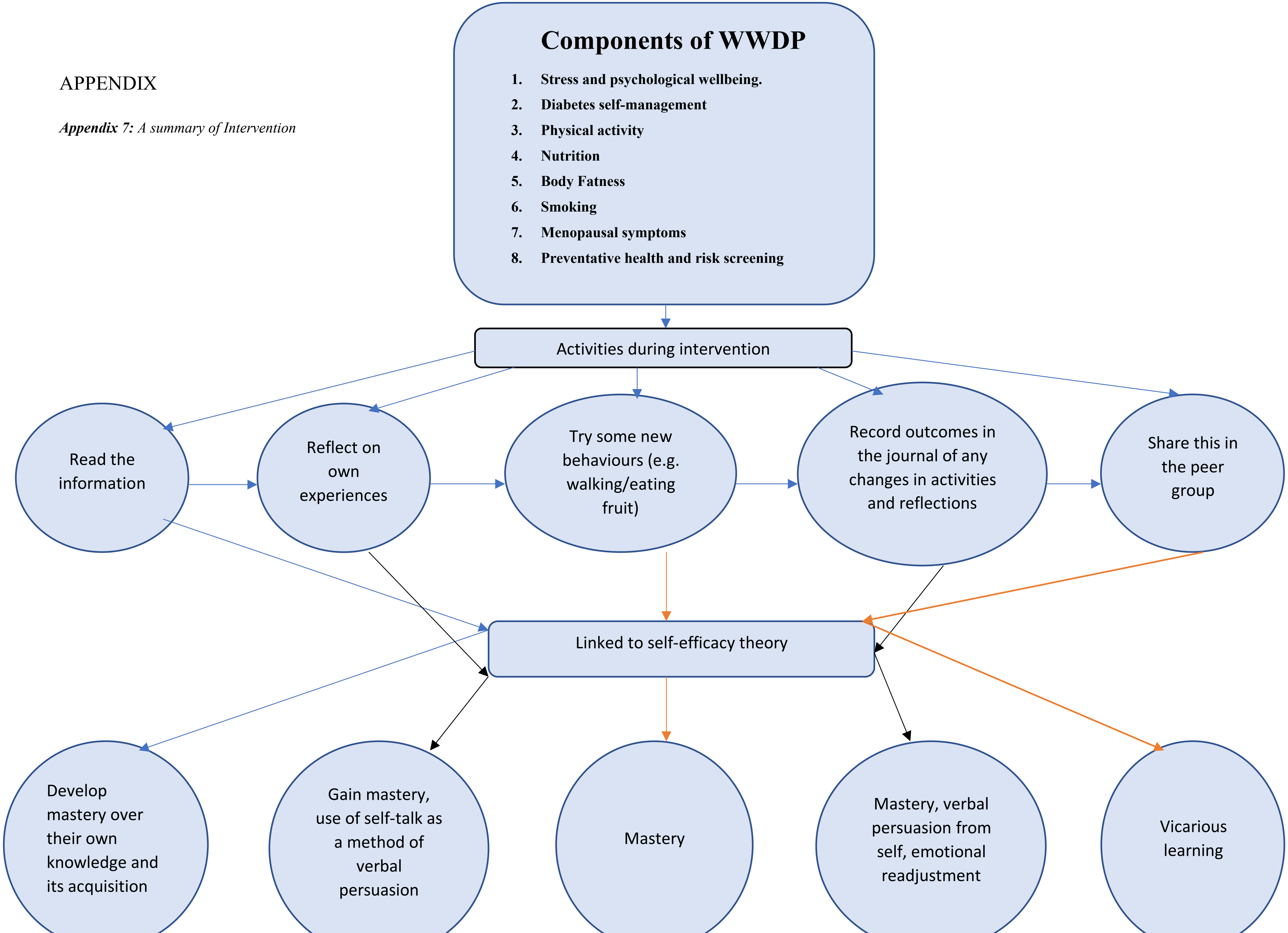

Appendix 8: WWDP PEER SUPPORT GROUP Handbook
