# Supplementary material for: The Women’s Wellness with Type 2 Diabetes Programme: Feasibility of an online peer support and goal-setting intervention for midlife women
Source: PLoS One. 2026 Mar 23;21(3):e0345517. doi: 10.1371/journal.pone.0345517 (PMC13008096; doi:10.1371/journal.pone.0345517)
Supplement: Attachment — Submitted filename: Response to Reviewers.docx [file pone.0345517.s008.docx]

The Academic Editor

PLOS ONE

13^th^ September 2025

Dear Dr Shairyzah Ahmad Hisham

We would like to express our sincere gratitude to you and the reviewers for your thoughtful and constructive feedback on our manuscript entitled " Integrating online peer support group and goal setting to promote behavioural changes in midlife women with type 2 diabetes: A feasibility study of the Women’s Wellness with Type 2 Diabetes Programme”.

We have carefully considered the reviewers' comments and have made revisions to address their concerns. In the table below, we provide detailed responses to each of the reviewers' comments alongside manuscript tracked changes file.

Thank you once again for your valuable feedback and support.

Deniz Bozkurt

Florence Nightingale Faculty of Nursing, Midwifery & Palliative Care

King’s College London

James Clerk Maxwell Building 5.40

London

| Reviewer 1 comment | Where addressed | Authors Response |
| --- | --- | --- |
| 1. Abstract. What’s WWDP? It would be better to attach your abbreviation when you spell them out for the first time before you use abbreviation in the text. | Abstract/Aim/Page 1 | Dear Reviewer, Thank you for noting this. Abbreviation has been added on line 15 after full use at first |
| 2. It might be informative to know whether there are other chronic diseases among the participants. | Table 2/Page 17 | Participants who have other chronic diseases are presented under diabetes complications in the table 2 on line 436-451. |
| 3. Line 160. Should be “from 1 to 10” | Method/Page 13 | It has been revised on new line 375-376. |
| 4. Paired t-test and Wilcoxon signed-rank test was implemented. It would be helpful to specify which variables were tested with which methods with normality assessment justification. | Method/Page 13 | Variables with method have been specified on line 392-394. |
| 5. Please clarify how the missing data were handled in the analysis. | Method/Page 14 | The clarification for missing data has been added on line 406-411. |
| 6. Self-report data were used. It would be helpful to discuss potential biases and their impact on the findings. | Discussion/Page 26 | Dear Reviewer, it has been discussed on line 594-597 as limitation. |
| 7.A scoring method was implemented, e.g., GAS. It would be helpful to explain how these scores were aggregated or what they represent. | Method/Page 13 | It has been explained on line 378-385. |
| 8. Please clarify how pre-specified thresholds were determined. | Method/Page 6 | The clarification has been added on line 157-161. |
| 9. As this is a feasibility study, it would be a great help to report or discuss how the resulting information serves as the resource for the future study, e.g., sample size planning. | Discussion/Page 25 | The discussion point has been added on line 569-573. |
| Reviewer 2 comment | Where addressed | Response |
| 1. The title does not represent the extent of research work. In this study, other than feasibility, there were other types of outcomes measured and reported in the results. I would suggest revising the title to better suit the actual research work done. | Title/Page 1 | Dear Reviewer, thank you for noting this. we have revised the title accordingly on line 1-3. |
| 2.Abstract: Aim  Consider simplifying the sentence. |  | Dear Reviewer, Thank you for the comment. |
| 3. Abstract: Methods  ▪To write the 1st sentence in full.  ▪To standardize the time frame used to weeks only.  ▪To add information on sampling method and methods used for group assignment.  ▪To add parameters/data collected to indicate feasibility. | Abstract/Methods | - It has been added on Line 19 Page 1. - Dear reviewer, thank you for the feedback. - This was a single-arm feasibility study; therefore, neither group assignment nor randomisation was conducted. - It has been added on line 21-22 Page 1. - The parameters are presented on line 22 Page 1. |
| 4.Abstract: Results  ▪The results stated are not directly related to the study aim. Consider revising this section.  ▪To include relevant results from statistical analyses used e.g., t-test etc. | Abstract/Results/Page 1-2 | - The aim has been revised accordingly on line 15-16 page 1. - The results have been added accordingly on line 33-42 page 2 |
| 5.Abstract: Other comments:  ▪To include 1-2 introductory lines before stating the study aim which could include introduction to the revised WWDP and study justification.  ▪To introduce the acronym WWDP when first mentioned in the study aim. | Abstract/Page 1 | - Dear reviewer, thank you for the feedback. - It has been added on line 15 page 1. |
| 6. Introduction: ▪Line 51-53: The statement on effect of menopause on hormonal changes is not applicable to all study participants as the study included women as young as 45 years of age. Please review this statement accordingly. | Introduction/Page 3 | The statement has been further justified on line 74-77. |
| 7. Introduction: ▪Consider adding operational definitions of major terms used in this study e.g., feasibility, effectiveness etc. | Introduction/Page 4 | The definitions have been added on line 105-108. |
| 8.Method: Design  ▪Line 81-82: It was not clear how feasibility was assessed at baseline. Please clarify. | Method/Page 8 | Dear reviewer, thank you for the feedback. The feasibility was assessed setting pre-defined criteria including recruitment rate and data completeness on line 297-306. |
| 9. Method: Study setting & recruitment  ▪Generally, I found the section could be further improved by re-arrangement of the sub-sections. For example, Ethics approval section would be best to be stated at the last part of this section and the Materials method could be brought earlier. | Method/Page 5 | Ethical permissions underpin the methods and; therefore, to support clarity, this detail has been moved to the beginning of the methods section, before participant recruitment. Line 120. |
| 10. Method: ▪Please clarify how recruitment was done using Google. | Method/Page 5 | The wording of the first sentence of recruitment section has been improved to clarify this. Line 128-129 |
| 11: Method: Line 100-101: It was unclear why only participants who scored > 2 in the DDS were included. In my opinion, this could be a source of bias that could affect the outcome on diabetes distress. Please clarify.  The exclusion criteria provided is automatic exclusion. Please revise accordingly. | Method/Page 6 | A sentence has been added to the end of the introduction signposting outcomes with important effect signals demonstrated by the previous feasibility study. Line 107-108.  A sentence has been added to the participant screening section of the methods to signpost that reductions in elevated diabetes distress was one of the outcomes with a strong signal and provides rationale for including women with elevated DD in this second study. Line 153-161. |
| 12. Method: It was not clear whether or not women who have undergone the WWDP program before, are still considered eligible to participate in this study. Please clarify. | Discussion/Page 26 | Women who had previously participated in the WWDP programme were not specifically excluded from this study. We did not collect data on this and we have added this as a limitation in the discussion on Line 591-593. |
| 13. Method: The groups and group assignment were not clearly stated. Assuming the word ‘intervention’ was used to refer to women underdoing the WWDP+ program, the ‘control’ group has to be explicitly stated. | Method/Page 5 | The study design section now states explicitly that this was a nonrandomised study and that all participants received the experimental intervention on Line 116-119. |
| 14. ▪I would recommend to begin reporting the total number of women participated in the study. | Results/Page 14 | It has been added on line 414. |
| 15. ▪I would recommend indicating the type of statistical analyses used as footnote in the Table 4. | Table 4/Page 23 | It has been added in table 4 as footnote on line 527-528 |
| 16. ▪ In the feasibility outcomes, it was stated that 82% of the participants completed the 3-month post baseline assessments. However, there was also the 6-month post assessment. It was not clear how many of these participants completed this assessment. Please clarify. | Results/Page 18 | It has been added on line 461-463. |
| 17. ▪ Figure 2 could have been more self-explanatory. I would also be interesting to report the potential reasons for the hikes and dips of the graphs. |  | Dear reviewer, Figure 2 shows participant engagement in the online Facebook private peer group. The reasons for peaks and dips in engagement will be explored in the qualitative analysis which is next phase of the study, where participants’ experiences and contextual factors influencing activity are discussed. |
| 18. ▪ Please state baseline and post assessments. | Results/Page 18 | It has been added on line 460-463. |
| 19. Discussion: ▪ I would like to recommend adding discussion points on how the improvement made to the program, compared to the previous version of WWDP, could make a difference to the studied population.  ▪ I would like to also recommend discussing potential confounding factors in this study. | Discussion/Page 24 | - It has been added on line 536-540. - A new paragraph has been added on line 576-579. |
| 20.Miscellaneous  ▪ Formatting: to rephrase sentences started with numbers e.g., Line 245 & 262. | Page 15 | New line number is 429-435 & 466, and they have been rephrased. |
| 21. Miscellaneous  ▪ In this study, there was no clear measurement of acceptance. Thus, it would not be relevant to conclude acceptability of the program. Please clarify. |  | Dear reviewer, we appreciate your review comments. Assessing through pre-defined progression criteria, feasibility targets were achieved: 77% recruitment of eligible women, 88% 12-week completion, and data completeness of 82% at 3 months and 79% at 6 months. Peer-group engagement met criterion (82% logged in ≥3 times). Goal setting was implementable and acceptable (88% set goals by week 3; 82% returned 12-week assessments; 68% achieved/exceeded expectations). Collection of clinical and psychosocial measures was feasible with high completion, yielding variability estimates to inform a future trial thus the program’s acceptability with above completion of pre-defined criteria. |
| Reviewer 3 comment | Where addressed | Response |
| 1. Interventions provided: The information on delivery of the content was only obtained when referring to the supplementary document. It should be briefly described under methods i.e. how long does it take for the participants to answer the baseline questions, by whom were the materials delivered and prepared, the flow of delivery by day etc. | Materials/Page 7 | Dear Reviewer, thank you for noting this. It has been added on line 189-195. |
| 2. Statistical software or programs used - SPSS? Jamovi? not indicated in the manuscript. | Method/Page 14 | The abbreviation has been added after full use on line 400. |
| 3. The interpretation of the discussion were not elaborated and sufficiently evidenced e.g. (Line 345) The sentence on limitations in achieving meaningful outcomes in physical activity was not explained and was left hanging. Important measures included in this clinical trial were BMI, HbA1c, glucose control. These measures are heavily associated with physical activity. An elaboration as to why it was not measured may provide justification on the design of the study. |  | Dear reviewer, we appreciate the opportunity to address your concerns regarding physical activity. Although improvements were observed in BMI and HbA1c, changes in physical activity were moderate. This may be explained by external factors influencing participants’ ability to engage in physical activity, such as life responsibilities or environmental barriers. For example, 25/34 participants were in full- or part-time employment, and 20 were married, which may have limited available time and energy for structured physical activity. The next phase, which is a qualitative study, will further explore participants’ experiences and contextual factors that may have influenced their engagement within programme components such as physical activity. We will ensure to explore external factors during next phase. |
| 4. Apart from that, line 329 stated that program fatigue, participant motivation or external influences may have reduced peer group engagement. Please include more explanation and evidences on this. The elaboration again will help to justify 'peer group engagement' as one of the intervention in improving T2DM among midlife women. | Discussion/Page 27 | The next phase of this study, which involves semi-structured interviews, will delve into the details of peer group engagement. The new line is 576-579. |
| 5. In supplementary document 2: Were the 215 posts came from participants only? It is understood that the facebook group is a private group. However, i am not sure whether the comments from the administrative personnel were included in the statistical analyses. There should also be some elaboration as to popular days and popular times for the comments to take place. This will help to strategize on when and how to approach participants appropriately. | Page 18 | It has been added on line 471-474. |
| 6. There is also a concern on traceability of the engagement of participants with the T2DM programme e-book. Was this documented during the study period? If not, perhaps this could be explained as part of the study limitation. Documentation on this is definitely important as it will affect the study outcomes. | Discussion/Page 26 | It has been added on line 580-584. |
| Editor comment | Where addressed | Response |
| 1. Please ensure that your manuscript meets PLOS ONE's style requirements, including those for file naming. The PLOS ONE style templates can be found at | Submission | Dear Editor, thank you for noting this. The file name has been changed. |
| 2. We note that you have selected “Clinical Trial” as your article type. PLOS ONE requires that all clinical trials are registered in an appropriate registry (the WHO list of approved registries is at. a) Please provide the complete date range for participant recruitment and follow-up in the methods section of your manuscript. b) If you have not yet registered your trial in an appropriate registry, we now require you to do so and will need confirmation of the trial registry number before we can pass your paper to the next stage of review. Please include in the Methods section of your paper your reasons for not registering this study before enrolment of participants started. Please confirm that all related trials are registered by stating: “The authors confirm that all ongoing and related trials for this drug/intervention are registered”. | Page 1 &5&6 | Dear Editor, both information is already presented on lines 48 &123-125 & 163-164. |
| 3. When completing the data availability statement of the submission form, you indicated that you will make your data available on acceptance. We strongly recommend all authors decide on a data sharing plan before acceptance, as the process can be lengthy and hold up publication timelines. Please note that, though access restrictions are acceptable now, your entire data will need to be made freely accessible if your manuscript is accepted for publication. This policy applies to all data except where public deposition would breach compliance with the protocol approved by your research ethics board. If you are unable to adhere to our open data policy, please kindly revise your statement to explain your reasoning and we will seek the editor's input on an exemption. Please be assured that, once you have provided your new statement, the assessment of your exemption will not hold up the peer review process. |  | Dear Editor, thank you for the feedback. Preparing the fully anonymised data will take some time, so it won’t be ready for this submission, but we will ensure it is available by acceptance. |
| 4. Please include your tables as part of your main manuscript and remove the individual files. Please note that supplementary tables (should remain/ be uploaded) as separate "supporting information" files”. |  | The tables are now presented in the main manuscript. |
| 5. Please remove all personal information, ensure that the data shared are in accordance with participant consent, and re-upload a fully anonymized data set.  Note: spreadsheet columns with personal information must be removed and not hidden as all hidden columns will appear in the published file. |  | They have been removed and fully anonymised. |
