# Supplementary material for: The Women’s Wellness with Type 2 Diabetes Programme: Feasibility of an online peer support and goal-setting intervention for midlife women
Source: PLoS One. 2026 Mar 23;21(3):e0345517. doi: 10.1371/journal.pone.0345517 (PMC13008096; doi:10.1371/journal.pone.0345517)
Supplement: Attachment — Submitted filename: Response_to_reviewers_auresp_2.docx [file pone.0345517.s010.docx]

| **Reviewer 2 comment** | **Where addressed** | **Response** |
| --- | --- | --- |
| 1. Abstract: Results  To consider omitting non-statistically significant results i.e., weight by 1.6kg | Abstract/Results | Dear Reviewer, we removed the only non-statistically significant result as recommended. Line 34. Thank you. |
| 2. Abstract: Conclusion  To state main conclusion of the study prior to stating the benefit of the results for future studies. | Abstract/Conclusion | Dear Reviewer, Thank you for your helpful feedback. We added a sentence in 40 and 41. |
| 3. Abstract: Other  Similar to previous suggestion, to include 1-2 introductory lines before stating the study aim which could include introduction to the revised WWDP and study justification. | Abstract/ Background | Dear Reviewer, thank you for your helpful feedback. We incorporated 2 (introduction/justification) sentences, replaced the title of aim within the Objective (Lines 13–20), as you recommended, while keeping the abstract within the word limit. |
| 4. Introduction: Line 85-87: The statements on study aim were inconsistent with the ones stated in the abstract. Please consider revising. | Introduction | Dear reviewer, we revised abstract's aim accordingly in Lines 18 to 20. |
| 5. Introduction: Similar to previous suggestions, consider adding operational definitions of major terms used in this study e.g., feasibility, effectiveness etc. | Introduction | Dear reviewer, we added operational terms for feasibility. Please see Lines 85 to 89. |
| 6. Materials and Method: Line 81-82: It was not clear how feasibility was assessed at baseline. Please clarify. | Design | Dear Reviewer, Line 81-82 was not included about the information, but we revised the referred sentence for feasibility recruitment rate at baseline in Line 162-163. |
| 7. Materials and Method: As previous suggestion, consider moving Ethics approval section to the last part of this section. | Ethics | Dear Reviewer, thank you for feedback and we moved the Ethics at the end of Method section. Please see 275-281 lines |
| 8. Materials and Method: As previous comment, the exclusion criteria provided is automatic exclusion. Please revise accordingly. |  | Dear Reviewer, thank you very much for constructive feedback and we revised the sentence accordingly. Please see Line: 132-134. |
| **Reviewer 3 Comment** | **Where addressed** | **Response** |
| 1.Overall the manuscript was well written. However, there are a few clarification required to further improve the manuscript. Under the 'Materials and methods: Study Setting and recruitment' subheading, the duration of disease (from the point of diagnosis) was not included as part of the inclusion criteria. From my point of view, the duration of disease will greatly affect the outcomes of study. If it is included in the DDS, it would be great if this can be clearly outlined in the paragraph. | Discussion | Dear Reviewer, Thank you for this helpful comment. We agree that duration of diabetes is an important characteristic that can influence study outcomes. In this feasibility study, duration of diabetes was not used as an inclusion criterion. This decision was informed by our patient and public involvement (PPI) study, where women highlighted that restricting eligibility based on years since diagnosis could unnecessarily limit participation and exclude relevant lived experiences. However, we did collect duration of diabetes at baseline as part of the demographic questionnaire. We have acknowledged this limitation in the Discussion section, which reads: *Duration of diabetes (years since diagnosis) was collected at baseline as part of the demographic questionnaire but was not used as an inclusion criteria which may have influenced participants’ responsiveness to the programme.* Line 435-438. |
| 2. As for the sample size, could you please detail out the recommendations by reference no 23 and 24 in order to reduce the level of uncertainty in determining the sample size. | Sample size | Dear Reviewer, we revised it accordingly. Please see 137-141 lines. |
| 3. Referring to table 2, the ethnicity of the sample population was not based on universal categories e.g., chinese should be under Asian. White Turkish is also not a universal ethnic group classification. The classification under others can further be detailed out in methods. | Table 2 | Dear Reviewer, Thank you for this constructive feedback. We revised it accordingly. Please see Table 2 and ethnicity categories. |
| 4. In table 2, you may want to arrange the 'self-reported diabetes treatment' according to treatment step up therapy. Example, 1st group should be diet and exercise followed by OHA and then OHA and insulin and so on so forth. With this we can screen through the sample population according to severity of disease. I would also like to clarify, all diabetic patients should be on diet, exercise and lifestyle modification. Perhaps you could consider reclassifying the samples. However, i am aware that this will affect some parts of your discussion and data presentation. | Table 2 | Dear Reviewer, we revised the Lines 288-290 accordingly and also revised the self-reported diabetes treatment in Table 2 as well.  Under table 2, lines 297-299 highlighted ** “Diabetes treatment was classified hierarchically, would be expected lifestyle advice including diet and exercise as part of usual care; therefore, the categories represent pharmacological treatment step-up.”* |
| 5. Similarly in table 2, the reported diabetes complications are not the universal diabetes complications as per stated in the guidelines i.e. macrovascular and microvascular complications including diabetic retinopathy, nephropathy etc. The listed diabetes complications are more of comorbidities. Kindly revise. | Table 2 | Dear Reviewer, Thank you very much for feedback. We separated diabetes complications and added column as other comorbid conditions in Table 2. |
| 6. Based on the above comments, there may be changes to the discussion part. Therefore i rest my further comments on the discussion part. All in all, this study offers beneficial outcomes in the management of diabetes among midlife women. Further studies should be conducted to cover a bigger sample population for better generalisability of the data. | Discussion | Dear reviewer, thank you for your feedback. We have added a sentence under Table 2 line 297-299 and discussion lines 388-390 and 435-438. |
