# Supplementary material for: The Women’s Wellness with Type 2 Diabetes Programme: Feasibility of an online peer support and goal-setting intervention for midlife women
Source: PLoS One. 2026 Mar 23;21(3):e0345517. doi: 10.1371/journal.pone.0345517 (PMC13008096; doi:10.1371/journal.pone.0345517)
Supplement: Attachment — Submitted filename: Response_to_reviewers_auresp_3.docx [file pone.0345517.s011.docx]

| **Reviewer 2 comment** | **Where addressed** | **Response** |
| --- | --- | --- |
| 1. Line 288, p15: To rephrase the sentence in order to prevent beginning it with a numeral. | Results | Dear Reviewer, we rephrased the sentence as recommended. Thank you.  Most participants had a family history of diabetes (n=25). |
| 2. Table 2, p 16-17: To double check the number (n) and percentage columns. A few variables in the number column were noted to be more than 34 and a few variables in the percentage column were noted have cumulative percentage of >100% (e.g., Marital status, education etc) or < 100% (e.g., Self-reported diabetes treatment etc.). Please correct accordingly. | Results/Table 2 | We thank the reviewer for the feedback. We have double checked and revised the percentages for single-response variables.  For comorbidities, frequencies and percentages exceed 100% as participants could select multiple options. A clarifying footnote has been added to the table 2. |
| 3. Line 351-352, p20: It was noted that the z value is missing for self-reported QoL. Please add accordingly. | Results | Dear Reviewer, Thank you very much for feedback. We added z value for QoL. (z=2.48; p <0.01) |
