# Supplementary material for: The Women’s Wellness with Type 2 Diabetes Programme: Feasibility of an online peer support and goal-setting intervention for midlife women
Source: PLoS One. 2026 Mar 23;21(3):e0345517. doi: 10.1371/journal.pone.0345517 (PMC13008096; doi:10.1371/journal.pone.0345517)
Supplement: Attachment — Submitted filename: PONE-D-25-24968 - Reviewer Comments.pdf [file pone.0345517.s005.pdf]

Manuscript title : Integrating Online Peer Support Group and Goal Setting to Promote Behavioural Changes in Midlife Women with Type 2 Diabetes: A feasibility study of the Women's Wellness with Type 2 Diabetes Programme

Comments/suggestions to authors for further improvement:

No.	Section in manuscript	Comments/suggestions
1.	Title	The title does not represent the extent of research work. In this study, other than feasibility, there were other types of outcomes measured and reported in the results. I would suggest revising the title to better suit the actual research work done.
2.	Abstract	**Aim**<br>Consider simplifying the sentence<br><br>**Methods**<br>▪ To write the 1st sentence in full.<br>▪ To standardize the time frame used to weeks only.<br>▪ To add information on sampling method and methods used for group assignment.<br>▪ To add parameters/data collected to indicate feasibility.<br><br>**Results**<br>▪ The results stated are not directly related to the study aim. Consider revising this section.<br>▪ To include relevant results from statistical analyses used e.g., t-test etc.<br><br>**Other comments:**<br>▪ To include 1-2 introductory lines before stating the study aim which could include introduction to the revised WWDP and study justification.<br>▪ To introduce the acronym WWDP when first mentioned in the study aim.
3.	Introduction	▪ Line 51-53: The statement on effect of menopause on hormonal changes is not applicable to all study participants as the study included women as young as 45 years of age. Please review this statement accordingly.<br>▪ Consider adding operational definitions of major terms used in this study e.g., feasibility, effectiveness etc.
4.	Materials and Method	**Design**<br>▪ Line 81-82: It was not clear how feasibility was assessed at baseline. Please clarify.<br><br>**Study setting & recruitment**<br>▪ Generally, I found the section could be further improved by re-arrangement of the sub-sections. For example, Ethics approval section would be best to be stated at the last part of this section and the Materials method could be brought earlier.<br>▪ Please clarify how recruitment was done using Google.

No.	Section in manuscript	Comments/suggestions
4.	Materials and Method (cont)	▪ Line 100-101: It was unclear why only participants who scored > 2 in the DDS were included. In my opinion, this could be a source of bias that could affect the outcome on diabetes distress. Please clarify. The exclusion criteria provided is automatic exclusion. Please revise accordingly.<br>▪ It was not clear whether or not women who have undergone the WWDP program before, are still considered eligible to participate in this study. Please clarify.<br>▪ The groups and group assignment were not clearly stated. Assuming the word 'intervention' was used to refer to women underdoing the WWDP+ program, the 'control' group has to be explicitly stated.
5.	Results	▪ I would recommend to begin reporting the total number of women participated in the study.<br>▪ In the feasibility outcomes, it was stated that 82% of the participants completed the 3-month post baseline assessments. However, there was also the 6-month post assessment. It was not clear how many of these participants completed this assessment. Please clarify.<br>▪ I would recommend indicating the type of statistical analyses used as footnote in the Table 4.<br>▪ The pre- and post-intervention analyses were not clear. The number of participants at each point was not similar i.e., baseline to post 3- and 6-month. I would recommend to clearly state the sets of comparisons e.g., between baseline and post 3 months and between baseline and post 6 months.<br>▪ Figure 2 could have been more self-explanatory. I would also be interesting to report the potential reasons for the hikes and dips of the graphs.
6.	Discussion	▪ I would like to recommend adding discussion points on how the improvement made to the program, compared to the previous version of WWDP, could make a difference to the studied population.<br>▪ I would like to also recommend discussing potential confounding factors in this study.
7.	Miscellaneous	▪ Formatting: to rephrase sentences started with numbers e.g., Line 245 & 262.<br>▪ In this study, there was no clear measurement of acceptance. Thus, it would not be relevant to conclude acceptability of the program. Please clarify.

Review completed on 25 June 2025
