# Supplementary material for: The Women’s Wellness with Type 2 Diabetes Programme: Feasibility of an online peer support and goal-setting intervention for midlife women
Source: PLoS One. 2026 Mar 23;21(3):e0345517. doi: 10.1371/journal.pone.0345517 (PMC13008096; doi:10.1371/journal.pone.0345517)
Supplement: Attachment — Submitted filename: PONE-D-25-24968 R1 - Reviewer Comments.pdf [file pone.0345517.s007.pdf]

PLOS-ONE Manuscript ID: PONE-D-25-24968-R1

Manuscript title : Feasibility of an Online Peer Support and Goal-Setting Intervention for Midlife Women

Comments/suggestions to authors for further improvement:

No.	Section in manuscript	Comments/suggestions
1.	Abstract	**Results**<br>▪ To consider omitting non-statistically significant results i.e., weight by 1.6kg<br><br>**Conclusion**<br>▪ To state main conclusion of the study prior to stating the benefit of the results for future studies.<br><br>**Other comments:**<br>▪ Similar to previous suggestion, to include 1-2 introductory lines before stating the study aim which could include introduction to the revised WWDP and study justification.
2.	Introduction	▪ Line 85-87: The statements on study aim were inconsistent with the ones stated in the abstract. Please consider revising.<br>▪ Similar to previous suggestions, consider adding operational definitions of major terms used in this study e.g., feasibility, effectiveness etc.
3.	Materials and Method	**Design**<br>▪ Line 81-82: It was not clear how feasibility was assessed at baseline. Please clarify.<br><br>**Study setting & recruitment**<br>▪ As previous suggestion, consider moving Ethics approval section to the last part of this section.<br>▪ As previous comment, the exclusion criteria provided is automatic exclusion. Please revise accordingly.

Review completed on 26 October 2025
