# Supplementary material for: The Women’s Wellness with Type 2 Diabetes Programme: Feasibility of an online peer support and goal-setting intervention for midlife women
Source: PLoS One. 2026 Mar 23;21(3):e0345517. doi: 10.1371/journal.pone.0345517 (PMC13008096; doi:10.1371/journal.pone.0345517)
Supplement: Attachment — Submitted filename: PONE-D-25-24968 R2 - Reviewer Comments.pdf [file pone.0345517.s009.pdf]

PLOS-ONE Manuscript ID: PONE-D-25-24968-R2

Manuscript title : Feasibility of an Online Peer Support and Goal-Setting Intervention for Midlife Women

Comments/suggestions to authors for further improvement:

No.	Section in manuscript	Comments/suggestions
1.	Results	**Baseline characteristics**<br>▪ Line 288, p15: To rephrase the sentence in order to prevent beginning it with a numeral.<br>▪ Table 2, p 16-17: To double check the number (n) and percentage columns. A few variables in the number column were noted to be more than 34 and a few variables in the percentage column were noted have cumulative percentage of >100% (e.g., Marital status, education etc) or < 100% (e.g., Self-reported diabetes treatment etc.). Please correct accordingly.<br><br>**Health-related outcomes findings**<br>▪ Line 351-352, p20: It was noted that the z value is missing for self-reported QoL. Please add accordingly.

Review completed on 8 January 2026
